# Lower- Versus Conventional-Sodium Dialysate and Left Ventricular Mass in Patients Receiving Hemodialysis: Causal Analyses of a Randomized Clinical Trial

**DOI:** 10.1016/j.xkme.2026.101417

**Published:** 2026-05-27

**Authors:** Mark R. Marshall, Alain C. Vandal, Christopher T. Chan, Janak R. de Zoysa, Ruvin S. Gabriel, Imad A. Haloob, Christopher J. Hood, Philip J. Matheson, David O.R. McGregor, Kannaiyan S. Rabindranath, John B.W. Schollum, David J. Semple, Zhengxiu Xie, Tian M. Ma, Joanna L. Dunlop

**Affiliations:** 1 Renal Service, Health NZ Waitematā, Auckland, New Zealand; 2 School of Medicine, Faculty of Medical and Health Sciences, University of Auckland, Auckland, New Zealand; 3 Department of Statistics, Faculty of Science, University of Auckland, Auckland, New Zealand; 4 Division of Nephrology, University Health Network, Toronto, ON, Canada; 5 Waitematā Clinical School, Faculty of Medical and Health Sciences, University of Auckland, Auckland, New Zealand; 6 Department of Cardiology, Middlemore Hospital, Counties Manukau Health, Auckland, New Zealand; 7 Department of Renal Medicine, Bathurst Base Hospital, New South Wales, Australia; 8 Department of Renal Medicine, Middlemore Hospital, Counties Manukau Health, Auckland, New Zealand; 9 Department of Nephrology, Wellington Regional Hospital, Health NZ Capital, Coast and Hutt Valley, Wellington, New Zealand; 10 Department of Nephrology, Christchurch Hospital, Health NZ Waitaha Canterbury, Christchurch, New Zealand; 11 Department of Nephrology, Waikato Hospital, Health NZ Waikato, Hamilton, New Zealand; 12 Nephrology Service, Dunedin Hospital, Health NZ Southern, Dunedin, New Zealand; 13 Department of Renal Medicine, Auckland City Hospital, Te Toka Tumai, Auckland, New Zealand; 14 Nutrition and Dietetics, Middlemore Hospital, Counties Manukau Health, Auckland, New Zealand; 15 Planning, Funding and Outcomes, Health New Zealand/Te Whatu Ora, Wellington, New Zealand

**Keywords:** Causal modeling, fluid overload, hemodialysis, hypertension, left ventricular hypertrophy, sodium

## Abstract

**Rationale & Objective:**

In a recent trial, allocation to lower dialysate [Na^+^] reduced interdialytic weight gain (IDWG), B-type natriuretic peptide (BNP) levels, and possibly extracellular fluid volume based on bioimpedance analysis. It did not, however, reduce left ventricular mass index (LVMI) as assessed by cardiac magnetic resonance imaging. This study aims to elucidate the cause of this unexpected finding.

**Study Design:**

We defined a causal pathway between allocation and LVMI, quantifying effects between physiological variables within the model.

**Setting & Participants:**

Home and self-care satellite hemodialysis patients.

**Exposure:**

Allocation to lower dialysate [Na^+^].

**Outcome:**

LVMI.

**Analytic Approach:**

We used structural equations for path analysis to estimate simultaneous effects of the main exposure on intermediary outcomes and intermediary outcomes on the main outcome.

**Results:**

The following latent constructs were identified as intermediary outcomes: “Fluid Volume” (indicated by N-terminal proBNP and BNP levels, overhydration index from bioimpedance analysis, and left ventricular end-diastolic volume from cardiac magnetic resonance imaging); “Dialytic BP” (indicated by pre-/postdialysis blood pressure [BP] measurements), “Interdialytic BP” (indicated by home/ambulatory BP measurements), antihypertensive dose, and IDWG. In the structural model, fluid volume had a large effect on LVMI (standardized path coefficient, 0.51; 95% CI, 0.35-0.67), and smaller ones on Dialytic BP and Interdialytic BP (respectively: 0.31; 95% CI, 0.15-0.47 and 0.22; 95% CI, 0.02-0.41). Dialytic BP and Interdialytic BP had small-to-medium-sized effects on LVMI (respectively: 0.23; 95% CI, 0.04-0.42 and 0.22; 95% CI, 0.07-0.37). Neither IDWG nor antihypertensive dose affected LVMI. Allocation to lower dialysate [Na^+^] had a large effect on IDWG (−0.37; 95% CI, −0.50 to −0.24), a marginal effect on antihypertensive dose (−0.20; 95% CI, −0.41 to 0.02), but none on fluid volume or BP constructs.

**Limitations:**

Allocation rather than the intervention itself; residual confounding from unrecorded intermediary outcomes.

**Conclusions:**

Allocation to lower dialysate [Na^+^] did not change LVMI because of insufficient effect on its main causes, namely fluid volume and BP. Although it reduced IDWG and antihypertensive dose, these factors did not causally affect LVMI.

## Plain-Language Summary

This study investigates why lowering sodium concentrations in dialysis fluid did not reduce heart muscle thickening during a recent clinical trial, although it reduced fluid gain between dialysis sessions and certain heart stress markers. We identified that heart muscle thickness is mainly caused by fluid excess in the body and high blood pressure (BP) and is not related to fluid gain between dialysis sessions. Although lower sodium dialysis successfully reduced fluid gain between sessions and possibly BP medication needs, it did not significantly reduce overall fluid excess in the body or BP—the key drivers of heart muscle thickening. Therefore, lower sodium dialysis simply was not strong enough to change the factors that truly matter: fluid excess in the body and BP.

## Graphical abstract

**Figure:**
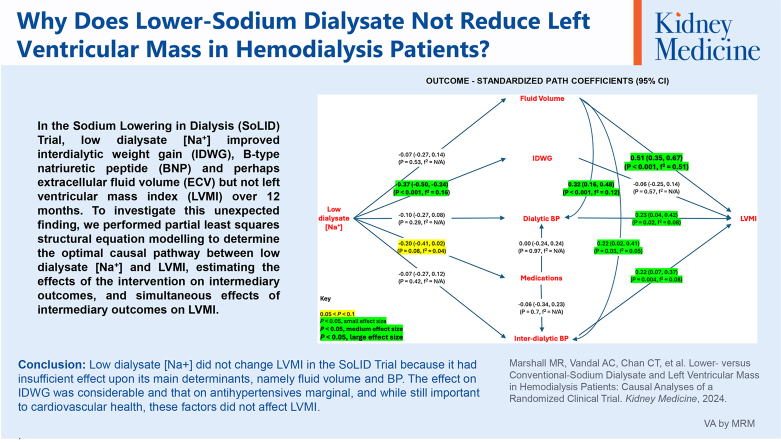

Reducing excess cardiovascular (CV) morbidity and mortality continues to be the leading unmet clinical need for patients on kidney replacement therapy. It has long been known that left ventricular (LV) hypertrophy (LVH) is highly prevalent in this population and a potent predictor of CV death.^1^^,^^2^ Moreover, LVH is considered a plausible causal factor on the pathway to CV death,^3^ because of well-established individual-level associations despite an absence of trial-level associations.^4^, ^5^, ^6^, ^7^, ^8^ One of the many purported contributors to LVH in this population is excessive body sodium content. The optimal removal of sodium-containing extracellular fluid by dialysis in a manner that avoids hypotension is the foundation of the “volume-first” approach, as espoused by opinion leaders in dialysis.^9^

One volume-first approach uses lower dialysate [Na^+^].^10^ Randomized controlled clinical trial evidence confirms reduced interdialytic weight gain (IDWG), antihypertensive medication use, and possibly blood pressure (BP).^11^ However, these benefits come at the cost of increased intradialytic hypotension and cramps, important prognostic and patient-centered outcomes.^12^, ^13^, ^14^, ^15^, ^16^, ^17^, ^18^, ^19^, ^20^ The net effect of lower dialysate [Na^+^] on CV mortality as a population health intervention is currently under study in an ongoing large cluster-randomized controlled trial.^21^

The effect of lower dialysate [Na^+^] on LV mass is informed by 2 randomized controlled trials.^22^^,^^23^ The larger and more methodologically robust is the Sodium Lowering in Dialysis (SoLID) trial (ACTRN12611000975998). In that trial, lower dialysate [Na^+^] did not reduce LV mass index (LVMI) assessed by cardiac magnetic resonance imaging (cMRI), despite improvements in secondary outcomes such as IDWG, B-type natriuretic peptide (BNP) levels, and possibly extracellular fluid volume (ECV). The present study explores these results through a causal framework to determine why the intervention did not improve LVMI, despite improving plausible intermediate outcomes on the causal pathway between dialysate [Na^+^] and LV mass. These questions were addressed through partial least squares structural equation modeling, a method that estimates complex cause-effect relationships.

## Methods

The SoLID trial was a multicenter, prospective, randomized, parallel group trial of lower compared with conventional dialysate [Na^+^] in home and self-care satellite hemodialysis (HD), conducted between January 2012 and March 2016 at 11 dialysis centers in New Zealand. Trial design, baseline characteristics, and the effects of the intervention on primary and several secondary outcomes have been published.^22^^,^^24^, ^25^, ^26^ Important exclusion criteria included: HD at a frequency >3.5 times per week, treatment with maintenance hemodiafiltration, sodium profiling during HD, documented infiltrative or hereditary cardiomyopathies, and important valve disease.

### Intervention

After randomization, patients assigned to lower dialysate [Na^+^] underwent an incremental decrease in dialysate [Na^+^] to 135 mM while staying on their otherwise usual dialysis prescription. In the lower dialysate [Na^+^] group, the corresponding procedure was undertaken to increase dialysate [Na^+^] to 140 mM. Allocation to lower dialysate sodium was considered the exposure of interest for the present study, defined in a binary manner (lower vs conventional dialysate [Na^+^]).

### Outcomes

cMRI was performed at baseline and 12 months using a standardized protocol; images were analyzed in a blinded fashion by 2 independent assessors at a central core laboratory. LVMI was considered the main outcome for the present study, defined as change in LVMI over 12 months.

### Other Measurements

During the 12-month intervention period, secondary outcomes were assessed at either quarterly intervals (IDWG, pre- and postdialytic BP, antihypertensive medication dose, plasma BNP levels, plasma N-terminal pro–B-type natriuretic peptide [NT-proBNP] levels, the overhydration index [defined as absolute overhydration in liters as a fraction of ECV divided by ECV/TBW {defined as ECV as a fraction of total body water}] determined using bioimpedance spectroscopy, predialysis serum [Na+]) or 6-monthly intervals (interdialytic BP). Procedural details of measurements and calculations can be found in the previous publications. Of note, we used IDWG rather than ultrafiltration volume, although these were very similar across the study with a high correlation (*r* = 0.9) with no significant difference between them on z-testing (Pr(|Z| > |z|) = 0.74). These secondary outcomes were modeled as potential indicators for the latent constructs as described below, computed as the change between measurements at 3 months onward compared with those at the study start (ie, *t* = 0), with final selection of these indicators contingent on subsequent model evaluation and optimization.

### Concise Methods

A detailed background on the statistical methods used for this study is provided in Item S1 (included as online supplementary material).

Analyses were conducted by partial least squares structural equation modeling, a statistical technique that analyzes the relationships between observed variables (variables that are measured directly) and latent constructs (variables that are not measured directly, but statistically inferred from a selection of observed, so-called indicator variables).

The main exposure in this study was allocation to lower dialysate [Na^+^] (observed variable from randomization) and the main outcome LVMI (observed measurement from cMRI). The variables on the causal pathway between the exposure and outcome were latent constructs, as shown in Table 1, with indicators being the secondary outcomes in the SoLID trial that fit their respective conceptual definitions. The constructs and indicators were sequentially refined into a final measurement model to ensure that the selection of indicators was accurate and representative. The criteria for final model selection included high face validity in a clinical sense, high correlation between indicators and their respective latent construct, no correlated errors, and no direct effect with other indicators.

**Table 1:** Description of Initial Variables Used in Structural Equation Modeling, Subject to Subsequent Evaluation and Refinement

Definition	Type of Variable	Indicator Variables
Lower dialysate [Na^+^]	Formative construct from observed measurement	Allocation
“Volume” - excess ECV in the body	Reflective latent construct	BNP (pmol/L), NT-proBNP (pmol/L), interdialytic weight gain (kg), LVEDV from cMRI (mL)∗∗, OHI, and ECV/TBW ratio from bioimpedance spectroscopy
“BP” – MAP pre- and postdialysis and in the interdialytic period at home	Latent construct	Predialysis MAP (mm Hg), postdialysis MAP (mm Hg), interdialytic MAP from home/ambulatory measurements (mm Hg)∗, predialysis serum [Na^+^] (mmol/L), antihypertensive medication (aggregated % of maximum recommended daily dose)
LVMI	Formative construct from observed measurement	LVMI

*Note:* 3-monthly measurements initially included as indicators except for.

Abbreviations: BNP, B-type natriuretic peptide; BP, blood pressure; ECV/TBW, extracellular fluid volume/total body water; IDWG, interdialytic weight gain; LVEDV, left ventricular end-diastolic volume; LVMI, left ventricular mass index; MAP, mean arterial pressure; NT-proBNP, N-terminal pro–B-type natriuretic peptide; OHI, overhydration index.

∗ signifying 6-monthly measurements and.

∗∗ signifying 12-monthly measurements.

After ensuring that the latent constructs were reliable and valid, we developed the structural model. We placed the latent constructs into a causal pathway starting with lower dialysate [Na^+^] allocation and finishing with LVMI, with an intervening structure informed by previous proposed causal pathways in the literature and our own clinical experience. After assessing robustness, we reported standardized parameter estimates and respective effect size.

A priori, we tested for effect moderation by baseline serum [Na^+^]. This is because of the Frequent Hemodialysis Network Trials, which demonstrated that patients with a higher baseline predialysis serum [Na^+^] had a greater reduction in LVMI with more frequent HD.^27^ We explored this question by undertaking group-specific modeling, with the subgroups defined as whether baseline predialysis serum [Na^+^] measurements were <138 mmol/L or ≥138 mmol/L. This was done instead of assessing a moderating effect of serum [Na^+^] on any specific step of the causal pathway, because of our uncertainty about where potential moderation might lie in the overall causal pathway.^28^

The presence of unobserved heterogeneity was assessed by exploring whether there were hidden subgroups within the model with different responses to allocation. Group-specific path modeling was then undertaken for more precise conclusions.

Statistical significance was set at a 0.05 level, although trends were acknowledged at a level of 0.10 to avoid type 2 errors, given the small sample size and exploratory nature of the analysis. Missing data were imputed using the k-nearest neighbors method.

We used an open-source implementation of the partial least squares structural equation modeling methodology in Stata 18 software for computations.^29^

## Results

### Patient Characteristics

A description of our patient sample is provided in Tables 2 and 3,^30^, ^31^, ^32^, ^33^, ^34^ reproduced with permission from published results of the SoLID trial.^22^

**Table 2:** Clinical Characteristics of SoLID Trial Sample, by Allocation (Reproduced With Permission^22^)

Characteristics	Dialysate [Na^+^] 135 mM	Dialysate [Na^+^] 140 mM	Total
No. (%) with data	49 (49%)	50 (51%)	99 (100%)
Age, y	51 (38-59)	51 (44-61)	51 (42-60)
Sex			
Male	34 (69%)	33 (66%)	67 (68%)
Female	15 (31%)	17 (34%)	32 (32%)
Vintage, y	2.0 (1-5)	2.0 (1.0-5.5)	2.0 (1-5)
Primary kidney disease			
Diabetic nephropathy	10 (20%)	16 (32%)	26 (26%)
Glomerulonephritis	23 (47%)	15 (30%)	38 (38%)
Other	16 (33%)	19 (38%)	35 (35%)
Diabetes mellitus	12 (24%)	19 (38%)	31 (31%)
Dialysis weekly hours			
≤18 h/wk	42 (86%)	45 (90%)	87 (88%)
>18 h/wk	7 (14 %)	5 (10%)	12 (12%)
Frequency of dialysis^a^			
Sessions/wk^a^	3.25 (0.42)	3.12 (0.24)	3.18 (0.34)
>3 sessions/wk	10 (20%)	8 (16%)	18 (18%)
Duration of dialysis^a^, h/session	5.1 (1.0)	4.7 (0.7)	4.9 (0.9)
Blood flow rate, mL/min	281 (41)	307 (30)	295 (38)
High-flux dialyzer	49 (100%)	50 (100%)	99 (100%)
Kt/V per session	1.38 (0.20)	1.42 (0.22)	1.40 (0.21)
stdKt/V per week	2.37 (0.37)	2.35 (0.28)	2.36 (0.31)
EKRc per week	15.1 (2.7)	15.2 (2.6)	15.2 (2.6)
Residual urine volume, mL/24 h	22.3 (0-405)	70.1 (0-446)	40.2 (0-437)
Anuria	23 (47%)	17 (34%)	40 (44%)
Dialysis setting			
Home	43 (88%)	39 (78%)	82 (83%)
Self-care satellite	6 (12%)	11 (22%)	17 (17%)
Ethnicity			
NZ Euro	22 (45%)	16 (32%)	38 (39%)
NZ Maori	9 (18%)	12 (24%)	21 (21%)
Asian	1 (2%)	4 (8%)	5 (5%)
Pacific peoples	15 (31%)	17 (34%)	32 (32%)
Other	2 (4%)	1 (2%)	3 (3%)
Body mass metrics			
Height, m	1.73 (0.1)	1.72 (0.1)	1.72 (0.1)
Weight, kg	87.2 (23.0)	89.7 (17.9)	88.5 (20.5)
Peripheral vascular disease	8 (16%)	5 (10%)	13 (13%)
Coronary artery disease	12 (24%)	10 (20%)	22 (22%)
Cerebrovascular disease	2 (4%)	2 (4%)	4 (4%)
Current smoker	8 (17%)	10 (20%)	18 (18%)
Serum albumin, g/L	37.5 (7.7)	37.6 (3.5)	37.5 (5.9)
Serum phosphate^a^, mmol/L	1.86 (0.55)	1.62 (0.43)	1.74 (0.51)
Serum calcium (uncorrected), mmol/L	2.33 (0.25)	2.37 (0.24)	2.35 (0.24)
Hemoglobin, g/L	116 (17)	111 (14)	113.0 (15.4)
Serum ferritin, μg/L	356 (299)	387 (277)	372 (287)
Transferrin saturation, %	23.7 (12.0)	25.1 (9.3)	24.4 (10.7)

Values are n (%), mean (standard deviation), or median (25%, 75% quartiles).

Abbreviations: EKRc, renal urea clearance; NZ, New Zealand; stdKt/V, standard Kt/V.

a *P* < 0.05. Cohen's d for frequency of dialysis, 0.38 (95% confidence interval, −0.02 to 0.78); Cohen's d for duration of dialysis, 0.46 (95% confidence interval, 0.06 to 0.86); and Cohen's d for serum phosphate, 0.49 (95% confidence interval, 0.09 to 0.89).

**Table 3:** Baseline Values of Study Outcomes by Allocation (Reproduced With Permission^22^)

Characteristics	Dialysate [Na^+^] 135 mM	Dialysate [Na^+^] 140 mM	Total
No. (%) with data	49 (49%)	51 (50%)	99 (100%)
cMRI parameters			
LVMI, g/m^2^	91.9 (25)	96.1 (27.4)	94.0 (26.2)
LVEDV, mL	190.5 (54.6)	196.2 (57.3)	193.4 (55.8)
LVESV, mL	83.8 (33.5)	88.3 (48.1)	86.1 (41.4)
Stroke volume, mL	106.6 (28.9)	107.9 (26.0)	107.3 (27.3)
Ejection fraction, %	56.9 (8.2)	56.9 (11.4)	56.9 (9.9)
Number with LVH^a^	27 (55%)	32 (64%)	59 (60%)
Interdialytic weight gain, kg	1.8 (0.9)	1.7 (0.8)	1.8 (0.8)
Interdialytic weight gain, % of predialysis weight	2.2 (1.1)	1.9 (0.8)	2.0 (1.0)
Bioimpedance analysis parameters, L			
Intracellular fluid	19.6 (4.7)	19.7 (3.8)	19.6 (4.2)
Extracellular fluid	21.5 (5.6)	21.4 (5.8)	21.4 (5.6)
Systolic blood pressure, mm Hg			
Interdialysis	144.3 (24.2)	146.9 (20.7)	145.6 (22.4)
Predialysis	144.6 (23.5)	149.3 (19.9)	146.9 (21.8)
Intradialysis	139.8 (23.2)	140.3 (18.6)	140.1 (20.9)
Postdialysis	140.1 (24.4)	142.4 (16.6)	141.3 (20.8)
Diastolic blood pressure, mm Hg			
Interdialysis	86.4 (14.8)	87.2 (10.4)	86.8 (12.7)
Predialysis	80.2 (13.5)	82.7 (13.3)	81.5 (31.4)
Intradialysis	79.7 (13.1)	78.5 (11.6)	79.1 (12.3)
Postdialysis	79.3 (15.5)	80.2 (11.4)	79.7 (13.5)
Mean arterial pressure, mm Hg			
Interdialysis	105.7 (16.9)	107.1 (12.9)	106.4 (14.9)
Predialysis	108.8 (15.0)	103.5 (14.4)	105.2 (13.8)
Intradialysis	99.7 (14.8)	99.2 (12.6)	99.4 (13.7)
Postdialysis	100 (17.1)	101.2 (11.4)	100.6 (14.5)
Dietary sodium intake, mg/d	2475 (901)	2531 (1024)	2502 (957)
Sodium mass removal^b^, mmol/dialysis session	288 (111)	220 (114)	263 (115)
KDQOL			
Burden of kidney disease	41 (28.2)	46.5 (27.3)	43.8 (27.7)
Effect of kidney disease	60 (24.9)	67.2 (20.4)	63.6 (22.9)
Symptoms of kidney disease	73.1 (16.1)	77.8 (16.5)	75.5 (16.4)
SF-36			
Mental Health Component	48.3 (10.8)	52.2 (8.8)	50.3 (10)
Physical Health Component	41.9 (8.1)	42.9 (9.3)	42.4 (8.7)
EQ5D score (of 100)	68.5 (17.9)	70.3 (19.6)	69.4 (18.7)
Xerostomia Inventory, score 5 to 25	11 (4.6)	10.6 (5)	10.8 (4.8)
Dialysis Thirst Inventory, score 7 to 35	18.2 (7.5)	17.4 (5.2)	17.8 (6.4)
Intradialytic hypotension, number of sessions (%)	16 (5.5%)	7 (2.4%)	23 (4.0%)
Predialysis plasma [Na+], mmol/L	138 (2.6)	138.3 (2.9)	138.2 (2.8)
Serum osmolality, mOsm/L	307.9 (10.9)	309.1 (8.6)	308.5 (9.8)
Plasma BNP, pmol/L	59 (87)	84.5 (203)	73 (164)
Plasma NT-proBNP, pmol/L	431 (919)	405 (1342)	431 (976)
Serum C-reactive protein, mg/L	8.4 (21.0)	7.6 (11.3)	8 (16.7)
Antihypertensive medications (aggregated % of maximum recommended daily dose), No. with 0 medications (median)	27 (0)	17 (24.4)	44 (11.9)

Values are n (%) or mean (standard deviation) unless otherwise specified.

Abbreviations: BNP, B-type natriuretic peptide; cMRI, cardiac magnetic resonance imaging; KDQOL, Kidney Disease Quality of Life survey; LVEDV, left ventricular end-diastolic volume; LVESV, left ventricular end-systolic volume; LVH, left ventricular hypertrophy; LVMI, left ventricular mass index; NT-proBNP, N-terminal pro–B-type natriuretic peptide; SF-36, 36-Item Short Form Health Survey.

a By criteria of Kawel-Boehm et al.^30^

b Sodium mass removed (ΔNa, in mmol) is calculated as ΔNa = (V_0_ × Na_0_) − (Vf × Naf), where V_0_ = predialysis total body water from bioimpedance analysis (liters); Na_0_ = predialysis serum sodium (mmol/L); Vf = postdialysis total body water from bioimpedance analysis (liters) = V_0_ − ultrafiltration volume; and Naf = postdialysis serum sodium (mmol/L). Analysis using variable volume single-pool model in 17 patients with available data.^31^, ^32^, ^33^, ^34^

### Primary and Secondary Outcomes

Table 4 shows the effects of lower dialysate [Na^+^] on the primary and selected secondary outcomes in the SoLID trial, reproduced with permission from published results of the trial. Dialysate [Na^+^] of 135 mM allocation did not result in a lower LVMI compared with 140 mM allocation but did result in a sustained decrease in IDWG, a marginal decrease in ECV, a sustained decrease in BNP, and a trend to reduction in antihypertensive medication. There was no difference in LV size and volumes between groups or in predialysis serum [Na+] between groups. Dialysate [Na^+^] of 135 mM allocation also resulted in increase in intradialytic hypotension, which was significant at 6 months and had a strong trend thereafter.

**Table 4:** Effect of Allocation to Dialysate [Na^+^] of 135 mM Relative to 140 mM on Selected Study Outcomes (Reproduced With Permission^22^)

Follow−up	3 mo	6 mo	9 mo	12 mo
**PRIMARY OUTCOME**				
LVMI (g/m^2^)	—	—	—	−3.94 (−10.52 to 2.63)
**SELECTED SECONDARY OUTCOMES**				
cMRI parameters				
LVEDV (mL)	—	—	—	−12.29 (−27.42 to 3.02)
LVESV (mL)	—	—	—	−9.5 (−21.88 to 2.88)
Stroke volume (mL)	—	—	—	−1.65 (−10.63 to 7.33)
Ejection fraction (%)	—	—	—	3 (−1 to 6)
Interdialytic weight gain (kg)	−0.47 (−0.80 to −0.15)^a^	−0.56 (−0.90 to −0.22)^a^	−0.90 (−1.26 to −0.54)^a^	−0.57 (−0.86 to −0.27)^a^
Interdialytic weight gain (% of predialysis body weight)	−0.52 (−0.87 to −0.17)^a^	−0.59 (−0.95 to −0.23)^a^	−0.93 (−0.13 to −0.52)^a^	−0.61 (−0.96 to −0.27)^a^
Extracellular fluid (L)	−1.05 (−1.81 to −0.30)^a^	−0.90 (−1.63 to −0.18)^a^	0.08 (−0.70 to 0.86)	−0.60 (−1.48 to 0.26)
Plasma BNP (ratio I/C)	0.74 (0.40-1.36)	0.43 (0.23-0.78)^a^	0.51 (0.28-0.94)^a^	0.49 (0.27-0.90)^a^
Plasma NT-proBNP (ratio I/C)	0.81 (0.41-1.61)	0.70 (0.35-1.39)	0.90 (0.45-1.80)	0.84 (0.42-1.68)
Mean arterial pressure (mm Hg)				
Predialysis	−1.11 (−5.76 to 3.52)	−2.94 (−7.49 to 1.61)	−0.71 (−5.56 to 4.14)	−0.92 (−6.51 to 4.68)
Intradialysis	−2.82 (−8.24 to 2.60)	−4.98 (−10.03 to 0.06)	−1.90 (−7.29 to 3.48)	1.08 (−5.02 to 7.18)
Postdialysis	−0.23 (−4.77 to 4.31)	−4.96 (−9.54 to −0.39)^a^	−0.70 (−5.69 to 4.28)	−1.23 (−7.04 to 4.59)
Interdialysis	—	0.89 (−4.35 to 6.14)	—	0.61 (−5.80 to 7.02)
Antihypertensive medications (aggregated fraction of maximum recommended daily dose)	—	—	—	−42.0 (−88.6 to 4.5)
Predialysis plasma [Na+] (mmol/L)	−1.2 (−2.4 to 0.1)	0.0 (−1.2 to 1.2)	−0.7 (−2.0 to 0.5)	0.4 (−0.8 to 1.6)
Intradialytic hypotension (OR)	1.5 (0.2-10.2)	7.5 (1.1-49.8)^a^	3.44 (0.5-23.6)	3.6 (0.5-28.8)

*Note:* Value are central estimates of effect (95% confidence interval), unless otherwise specified.

Abbreviations: BNP, B-type natriuretic peptide; cMRI, cardiac magnetic resonance imaging; I/C, intervention/control; LVEDV, left ventricular end-diastolic volume; LVESV, left ventricular end-systolic volume; LVMI, left ventricular mass index; NT-proBNP, N-terminal pro–B-type natriuretic peptide; OR, odds ratio.

a *P* < 0.05.

### Measurement Model

The initial measurement model before evaluation and refinement is shown in Table 5 and the final optimized model in Table 6.

**Table 5:** Initial Measurement Model—Standardized Loadings and Assessments of Internal Consistency and Construct Reliability

	LVMI	Lower Dialysate [Na^+^] Allocation	BP	Volume
Change in LVMI at 12 mo	1.000	—	—	—
Dialysate [Na^+^] allocation	—	1.000	—	—
Change in serum [Na^+^] at 3 mo	—	—	0.185	—
Change in serum [Na^+^] at 6 mo	—	—	0.141	—
Change in serum [Na^+^] at 9 mo	—	—	0.344	—
Change in serum [Na^+^] at 12 mo	—	—	0.220	—
Change in home dialysis MAP at 6 mo	—	—	0.600	—
Change in home dialysis MAP at 12 mo	—	—	0.548	—
Change in predialysis MAP at 3 mo	—	—	0.404	—
Change in predialysis MAP at 6 mo	—	—	0.697	—
Change in predialysis MAP at 9 mo	—	—	0.739	—
Change in predialysis MAP at 12 mo	—	—	0.661	—
Change in postdialysis MAP at 3 mo	—	—	0.523	—
Change in postdialysis MAP at 6 mo	—	—	0.743	—
Change in postdialysis MAP at 9 mo	—	—	0.798	—
Change in postdialysis MAP at 12 mo	—	—	0.709	—
Change in antihypertensive medications at 3 mo	—	—	0.147	—
Change in antihypertensive medications at 6 mo	—	—	0.187	—
Change in antihypertensive medications at 9 mo	—	—	0.283	—
Change in antihypertensive medications at 12 mo	—	—	0.313	—
Change in LVEDV at 12 mo	—	—	—	0.678
Change in serum NT-proBNP at 3 mo	—	—	—	0.262
Change in serum NT-proBNP at 6 mo	—	—	—	0.720
Change in serum NT-proBNP at 9 m	—	—	—	0.822
Change in serum NT-proBNP at 12 mo	—	—	—	0.817
Change in serum BNP at 3 mo	—	—	—	0.478
Change in serum BNP at 6 mo	—	—	—	0.700
Change in serum BNP at 9 mo	—	—	—	0.824
Change in serum BNP at 12 mo	—	—	—	0.854
Change in OHI at 3 mo	—	—	—	0.499
Change in OHI at 6 mo	—	—	—	0.522
Change in OHI at 9 mo	—	—	—	0.596
Change in OHI at 12 mo	—	—	—	0.616
Change in ECV/TBW at 3 mo	—	—	—	0.449
Change in ECV/TBW at 6 mo	—	—	—	0.402
Change in ECV/TBW at 9 mo	—	—	—	0.474
Change in ECV/TBW at 12 mo	—	—	—	0.342
Change in IDWG at 3 mo	—	—	—	-0.201
Change in IDWG at 6 mo	—	—	—	-0.180
Change in IDWG at 9 mo	—	—	—	-0.081
Change in IDWG at 12 mo	—	—	—	-0.066
Cronbach’s alpha	N/A	N/A	0.812	0.860
Dillon-Goldstein’s rho	N/A	N/A	0.836	0.863
Dijkstra-Henseler’s rho	N/A	N/A	0.870	0.947
Average variance extracted	N/A	N/A	0.260	0.313
Heterotrait–monotrait ratio	N/A	N/A	0.156	

Abbreviations: BNP, B-type natriuretic peptide; BP, blood pressure; ECV/TBW, extracellular fluid volume/total body water; IDWG, interdialytic weight gain; LVEDV, left ventricular end-diastolic volume; LVMI, left ventricular mass index; MAP, mean arterial pressure; N/A, not applicable; NT-proBNP, N-terminal pro–B-type natriuretic peptide; OHI, overhydration index.

**Table 6:** Final Measurement Model—Standardized Loadings and Assessments of Internal Consistency and Construct Reliability

	LVMI	Lower Dialysate [Na^+^] Allocation	Interdialytic BP	Dialytic BP	Medications	Volume	IDWG
Change in LVMI at 12 mo	1.000	—	—	—	—	—	—
Dialysate [Na^+^] allocation	—	1.000	—	—	—	—	—
Change in home dialysis MAP at 6 mo	—	—	0.861	—	—	—	—
Change in home dialysis MAP at 12 mo	—	—	0.869	—	—	—	—
Change in predialysis MAP at 6 mo	—	—	—	0.703	—	—	—
Change in predialysis MAP at 9 mo	—	—	—	0.818	—	—	—
Change in predialysis MAP at 12 mo	—	—	—	0.693	—	—	—
Change in postdialysis MAP at 6 mo	—	—	—	0.752	—	—	—
Change in postdialysis MAP at 9 mo	—	—	—	0.864	—	—	—
Change in postdialysis MAP at 12 mo	—	—	—	0.768	—	—	—
Change in antihypertensive medications at 3 mo	—	—	—	—	0.844	—	—
Change in antihypertensive medications at 6 mo	—	—	—	—	0.948	—	—
Change in antihypertensive medications at 9 mo	—	—	—	—	0.911	—	—
Change in antihypertensive medications at 12 mo	—	—	—	—	0.684	—	—
Change in LVEDV at 12 mo	—	—	—	—	—	0.697	—
Change in serum NT-proBNP at 6 mo	—	—	—	—	—	0.735	—
Change in serum NT-proBNP at 9 mo	—	—	—	—	—	0.844	—
Change in serum NT-proBNP at 12 mo	—	—	—	—	—	0.832	—
Change in serum BNP at 6 mo	—	—	—	—	—	0.718	—
Change in serum BNP at 9 mo	—	—	—	—	—	0.859	—
Change in serum BNP at 12 mo	—	—	—	—	—	0.888	—
Change in OHI at 6 mo	—	—	—	—	—	0.445	—
Change in OHI at 9 mo	—	—	—	—	—	0.573	—
Change in OHI at 12 mo	—	—	—	—	—	0.579	—
Change in IDWG at 3 mo	—	—	—	—	—	—	0.709
Change in IDWG at 6 mo	—	—	—	—	—	—	0.811
Change in IDWG at 9 mo	—	—	—	—	—	—	0.843
Change in IDWG at 12 mo	—	—	—	—	—	—	0.806
Cronbach’s alpha	N/A	N/A	0.664	0.864	0.883	0.899	0.804
Dillon-Goldstein’s rho	N/A	N/A	0.856	0.896	0.913	0.917	0.872
Dijkstra-Henseler’s rho	N/A	N/A	0.665	0.894	0.930	0.940	0.820
Average variance extracted	N/A	N/A	0.749	0.591	0.727	0.534	0.630
Heterotrait–monotrait ratio	N/A	N/A	<0.695				

Abbreviations: BNP, B-type natriuretic peptide; BP, blood pressure; ECV/TBW, extracellular fluid volume/total body water; IDWG, interdialytic weight gain; LVEDV, left ventricular end-diastolic volume; LVMI, left ventricular mass index; NT-proBNP, N-terminal pro–B-type natriuretic peptide; MAP, mean arterial pressure; N/A, not applicable; OHI, overhydration index.

#### Blood Pressure

Several refinements were made in refining to the initial “BP” measurement model before arriving at the final specification. Serum [Na^+^] and antihypertensive medication were removed as indicators because of low factor loadings. However, because of the clinical relevance of medication to CV health in general, these indicators were formed into new latent construct called “Medications” to be included separately in the structural model. In addition, the loadings of interdialytic ambulatory/home BP were consistently below 0.6 when these measurements were combined with pre- and postdialysis BP in a single construct. A separate latent construct was therefore defined as “Interdialytic BP” indicated by ambulatory/home BP measurements, and the original BP construct redefined as being “Dialytic BP,” indicated by only pre- and postdialysis BP measurements. Finally, 3-month pre- and postdialysis BP continued to have loadings well below 0.6 during model refinement and were therefore removed as indicators.

The evolution of the optimized latent constructs of Interdialytic BP, Dialytic BP, and Medications is provided in detail in Table S1 (included as online supplementary material). As can be seen, stepwise model refinement improved indicator loadings, internal consistency, and convergent validity to an acceptable standard while maintaining discriminant validity. Constructs met criteria for unidimensionality with a single factor having an eigenvalue >1 (Interdialytic BP, 1.6; Dialytic BP, 3.5).

#### Volume

Several refinements were also made to the “Volume” measurement model. IDWG measurements were removed as indicators because of low factor loadings but formed into new latent construct defined as “IDWG” to be included separately in the structural model. Three-month indicators for BNP and NT-proBNP were also removed because of low factor loadings. Of the 2 bioimpedance spectroscopy indices, overhydration index is a more direct assessment of fluid overload compared with ECV/TBW ratio. The latter was therefore removed from the model, further justified by its relatively lower loadings. Finally, 3-month and 6-month overhydration index indicators continued to have low factor loadings well below 0.6 and were therefore removed as indicators while retaining those at 9 and 12 months.

The evolution of the initial volume latent construct is provided in detail in Table S2 (included as online supplementary material), along with model performance metrics showing good validity. Constructs met the criteria for unidimensionality (eigenvalue for volume = 5.9, IDWG = 2.7).

### Main Structural Model

**Figure 1:**
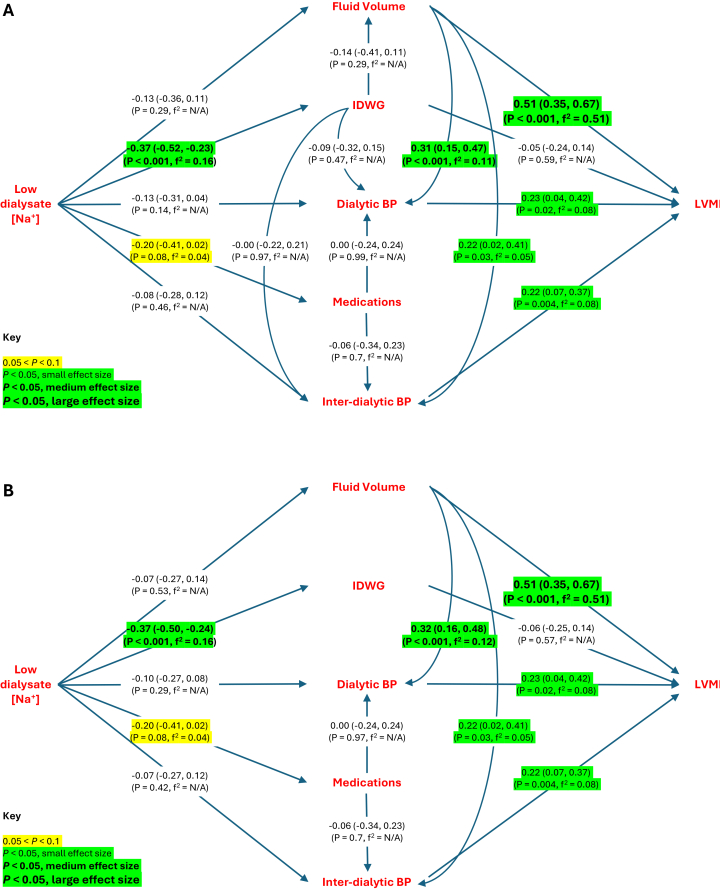
(a) Initial main structural model of causal effects. (b) Final main structural model of causal effects. Abbreviations: BP, blood pressure; IDWG, interdialytic weight gain; LVMI, left ventricular mass index; N/A, not applicable.

The main structural model is shown in Fig 1. Effects within the model are reported as standardized parameter estimates, interpreted as the number of standard deviations by which the outcome mean increases for every 1 standard deviation increase of the predictor variable. The size of the respective effects were classified as being small, medium, and large, based on β values and Cohen’s *f*^2^.

The effects of IDWG on volume and BP were negligible as shown in Fig 1a, and therefore, these paths were removed from the final model as shown in Fig 1b. The *R*^2^ for LVMI was 0.53, indicating that around half the variation in LVMI was explained by the model. However, the *R*^2^ for the other dependent variables varied from 0.01-0.13, indicating smaller explanatory power. Technically, overall model fit was adequate but mediocre, with an average *R*^2^ for the entire model of 0.15 and a standardized root mean square residual of 0.12. The highest variance inflation factor associated with the model was 1.5, indicating unlikely collinearity. *Q*^2^ values varied between 0.01 and 0.04, implying some, but not high, predictive relevance.

Examining the global model for insights, it can be inferred that volume had a large direct effect on LVMI, and small-to-medium-sized indirect effect mediated through BP. Interdialytic BP and Dialytic BP both had small-to-medium-sized direct effects on LVMI.

Lower dialysate [Na^+^] allocation had a medium-to-large effect on IDWG, which in turn had no direct or indirect effects on LVMI. Allocation had no effect on volume or either BP construct, although it had a trend toward reducing antihypertensive medication dose. Of note, antihypertensive medication dose had no effect on BP. Although not included in the structural model as initially proposed, sensitivity testing showed that antihypertensive medication dose had no direct effect on LVMI (*P* = 0.18).

**Figure 2:**
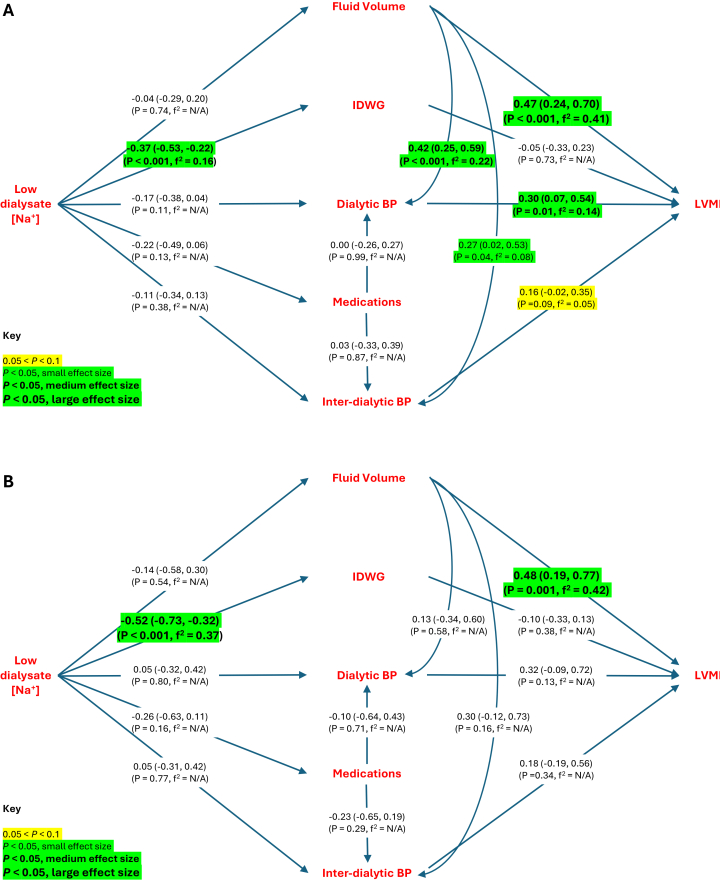
(a) Structural model in the subgroup with baseline serum [Na^+^] ≥138 mmol/L. (b) Structural model in the subgroup with baseline serum [Na^+^] <138 mmol/L. Abbreviations: BP, blood pressure; IDWG, interdialytic weight gain; LVMI, left ventricular mass index; N/A, not applicable.

Potential for moderation by baseline predialysis serum [Na^+^] is shown in Fig 2. In the subgroup with lower serum [Na^+^], the effect of volume on Dialytic BP is nonsignificant, as compared with having a medium-to-large effect in the other subgroup. The effects of BP on LVMI were also less certain. Other point estimates remain similar in direction and magnitude in both subgroups.

### Unobserved Heterogeneity in Main Structural Model

A detailed description of unobserved heterogeneity is provided in Tables S3 and S4 (included as online supplementary material). Testing showed that there are at least 2 homogenous groups within the data with potentially different model fit within them. Group-specific path models are shown in Fig S1 (included as online supplementary material). The core characteristics of the model are the same for each subgroup, namely that: Volume has a large effect on LVMI and lower dialysate [Na^+^] allocation has a medium-to-large effect on IDWG, which in turn does not affect LVMI. However, the groups differed in how lower dialysate [Na^+^] allocation affects antihypertensive medication and how volume affects BP. Further characterization of the identified subgroups is planned but out of scope for this article.

## Discussion

There are 5 groups of insights that arise from these analyses.

The first group pertains to the primary outcome of LVMI. Around half of the observed variation in LVMI in the SoLID trial can be explained by volume and BP. This means that around 50% of the variation in LVMI was due to factors other than volume and BP, perhaps anemia, abnormalities in mineral metabolism, and neurohormonal factors.^35^, ^36^, ^37^, ^38^, ^39^, ^40^, ^41^, ^42^ Notwithstanding, addressing volume and BP can be expected to have a large effect on LVH in this population, although the low factor loadings at 3 and 6 months within both constructs suggest that changes in volume and BP status have effects on LVMI only after several months of exposure and that clinically significant effects might take years to develop. Overall, however, study analyses support the volume-first paradigm^9^^,^^43^^,^^44^ and also reinforces randomized controlled trial data identifying BP as a major therapeutic target for reducing CV mortality in dialysis patients.^45^

A second group of insights concerns volume status, which was best assessed as a combination of BNP/NT-proBNP and bioimpedance analysis parameters. We found that IDWG was very poorly reflective of volume status, going against clinical heuristics that have customarily included IDWG as a part of volume management. Our finding is consistent with another clinical trial that also examined this question.^35^ Of note, IDWG did not have any relationship with BP in the SoLID trial, despite having a modest role in BP increases in another study.^46^ This discrepancy probably arises from our particularly careful approach to quantifying patient volume status. Our conclusion should not minimize the clinical importance of IDWG, because it is coupled to ultrafiltration rate, which is an independent risk factor for mortality.^47^^,^^48^ However, this effect is most likely mediated through the mechanism of recurrent intradialytic hypotension,^16^^,^^49^, ^50^, ^51^ with little or no mediation through increased LVH. Another more minor nuance within this group of findings is that overhydration index was a relatively better reflection of volume compared with ECV/TBW ratio, perhaps because of its greater directness.^52^^,^^53^ Overall, this group of insights suggests that, despite contradictory opinions in the literature, neurohormonal biomarkers and bioimpedance analysis should be considered as routine assessments of volume status of HD patients.^54^, ^55^, ^56^, ^57^, ^58^, ^59^, ^60^, ^61^

A third group of insights concerns BP, which, in the SoLID trial, was best assessed as interdialytic and pre-/postdialytic BP separately.^62^, ^63^, ^64^, ^65^, ^66^ These clinical measurements did not reflect each other well, with separate and similarly sized effects in the model that were independent. Somewhat counterintuitively, neither BP construct was affected by the presence and extent of antihypertensive medication, although, as expected, there was a small-to-medium sized effect of fluid volume on BP. Our findings suggest that home or ambulatory BP measurements should also be routine assessments in optimizing CV health of HD patients, in addition to the usual pre- and postdialysis ones.

The fourth group of insights concerns the role of predialysis serum [Na^+^]. In our subgroup analysis, we found that the effects of BP on LVMI were more prominent in those with a higher rather than lower serum [Na^+^], suggesting that any intervention to reduce BP might have a less consistent effect in those with a low predialysis serum [Na^+^]. Looking forward, studies of dialysis interventions to reduce BP might be best to select or stratify on this patient characteristic, focusing on a possibly more sensitive population characterized by a higher predialysis serum [Na^+^].

The final group of insights concerns our key clinical question: why does lower dialysate [Na^+^] not improve LVMI, despite improving IDWG, BNP, and ECV? In the SoLID trial (as well as other trials), lower dialysate [Na^+^] allocation had a large effect on reducing IDWG. However, as we noted in this analysis, isolated change in IDWG had no direct or indirect effect on LVMI, consistent with other clinical trial data as mentioned above.^35^ The effects of lower dialysate [Na^+^] on fluid volume in the SoLID trial were minimal, a crucial finding given the finding that fluid volume had the strongest causal effect on LVMI in the trial. Moreover, the effects of lower dialysate [Na^+^] allocation on BP in the SoLID trial were minimal, although there was a trend to reduced antihypertensive medication. It is important to distinguish between the effects of BP and antihypertensive medication in the SoLID trial; the structural model showed that if BP is lower, then LVMI is also lower, whereas a change in antihypertensive medication has no effect on LVMI. The combination of these findings suggests that clinicians in the SoLID trial responded to the effects of lower dialysate [Na^+^] on BP by reducing antihypertensive medication, rather than allowing the BP to decrease. This was probably driven by the increase in intradialytic hypotension that was observed in the group allocated to lower dialysate [Na^+^]. We speculate that if a different practice pattern had been observed, whereby the BP was allowed to drop with antihypertensive medications remaining the same (albeit in a safe manner, for instance, by increasing dialysis session length), there may have been an observed change in LVMI mediated through the change in BP. This would be consistent with the observations of others, albeit using dialysis interventions other than the one used in this study.^35^^,^^67^

The main strength of this analysis is the study design, with randomization that removes all confounding between dialysate [Na+] allocation and LVMI, and the use of structural equation modeling for examining causal pathways anchored at each end by these elements. This strategy has traditionally been used in the behavioral sciences but is increasingly used to explore potentially causal mediating relationships between physiological and clinical variables.^68^, ^69^, ^70^, ^71^, ^72^, ^73^, ^74^ Identifying the most plausible chain of events leading to outcomes and the mode of action on any given intervention can inform future research directions and allow the development of more nuanced quality improvement initiatives with a higher chance of having positive impacts.

There are several weaknesses of the study. The first is that we modeled only the effect of lower dialysate [Na+] allocation, not intervention. There were over 130 single episodes of wrong dialysate [Na^+^] utilization involving over half the participants in the study. It would be possible to explore the effect of treatment rather than allocation, although this exercise is out of scope for this article. The second pertains to the speculative nature of our causal pathway, although the general form of the pathway, while parsimonious, is consistent with current customary thought and paradigms in the area.^2^^,^^3^^,^^49^^,^^75^^,^^76^ Nonetheless, we did not include confounders that might also affect LVMI or indeed function as mediators (or moderators) in our currently proposed causal pathway, such as such as neurohormonal biomarkers.^1^^,^^37^, ^38^, ^39^ A third weakness is that we modeled BP as the mean arterial pressure, omitting the potential effect of pulse pressure on outcomes.^77^, ^78^, ^79^ Fourth, we did not separately model classes of antihypertensive medications that may have differential effects on LVMI independent of BP, such as renin angiotensin system blockers. Fifth, the home HD population in this study may have a different response to therapy compared with conventional facility or peritoneal dialysis patients, limiting extrapolation of findings. Finally, our measurement model was perhaps less than ideal, with very good internal consistency, moderate-to-good convergence validity, but mediocre discriminant validity.

In summary, under the structural model considered, lower dialysate [Na^+^] allocation had no effect on LVMI in the SoLID trial likely because of insufficient effect on its main determinants, namely volume and BP. The effect of lower dialysate [Na^+^] allocation on IDWG was considerable and the effect on antihypertensive medication marginal. Although these latter effects are of relevance to CV health through mechanisms such as dialysis-associated circulatory stress, there is no evidence in our study that they affect LVMI. Further analyses are currently underway that will analyze the effect of the intervention on markers of myocardial microinjury (eg, troponin T, cMRI wall motion score index, T1 mapping) and the association between dialysate–plasma [Na^+^] gradient and primary and secondary outcomes in the trial.

## Supplementary Materials

Supplementary (PDF) Table S1-S4; Figure S1a-b; Item S1
