# Supplementary material for: Lower- Versus Conventional-Sodium Dialysate and Left Ventricular Mass in Patients Receiving Hemodialysis: Causal Analyses of a Randomized Clinical Trial
Source: Kidney Med. 2026 May 27;8(9):101417. doi: 10.1016/j.xkme.2026.101417 (PMC13485629; doi:10.1016/j.xkme.2026.101417)
Supplement: Supplementary file 1 [file mmc1.pdf]

Supplementary Material

Table of Contents

Table of Contents **1**

Supplementary Tables **2**

Table S1 - Evolution of the initial "BP" latent construct into the final latent constructs of "Dialytic BP" and "Inter-dialytic BP" for measurement model *3*

Table S2 - Evolution of the initial latent construct of "Fluid Volume" into the final latent constructs of "Fluid Volume" and "IDWG" for the measurement model *4*

Table S3 - Finite mixture partial least squares (FIMIX-PLS) comparison for the structural model *6*

Table S4 – PLS-GAS comparison for the structural model *7*

Supplementary Figures **8**

Figure S1a. Structural model in GAS 1 subgroup *9*

Figure S1b. Structural model in GAS 2 subgroup *10*

Appendix 1 - Detailed Statistical Methods **11**

Introduction to various approaches to structural equation modelling (SEM) *11*

Overall approach to structural equation modelling in this study *16*

Detailed methods for building the measurement model in this study *21*

Detailed methods for building the structural models in this study *22*

Supplementary References **24**

Supplementary Tables

Table S1 - Evolution of the initial “BP” latent construct into the final latent constructs of “Dialytic BP” and “Inter-dialytic BP” for measurement model

	BP	BP	Inter-dialytic BP	Dialytic BP	Interdialytic BP	Dialytic BP
	Initial	After removal of serum [Na^+]	After removal of antihypertensive medications, and separation into two constructs	After removal of antihypertensive medications, and separation into two constructs	After removal 3 month indicators from Dialytic BP	After removal 3 month indicators from Dialytic BP
Change in serum [Na^+] at 3 months	0.185					
Change in serum [Na^+] at 6 months	0.141					
Change in serum [Na^+] at 9 months	0.344					
Change in serum [Na^+] at 12 months	0.220					
Change in home dialysis MAP at 6 months	0.600	0.609	0.895		0.898	
Change in home dialysis MAP at 12 months	0.548	0.545	0.830		0.833	
Change in pre-dialysis MAP at 3 months	0.404	0.401		0.488		
Change in pre-dialysis MAP at 6 months	0.697	0.695		0.744		0.719
Change in pre-dialysis MAP at 9 months	0.739	0.76		0.826		0.829
Change in pre-dialysis MAP at 12 months	0.661	0.642		0.699		0.711
Change in post-dialysis MAP at 3 months	0.523	0.537		0.584		
Change in post-dialysis MAP at 6 months	0.743	0.741		0.765		0.748
Change in post-dialysis MAP at 9 months	0.798	0.821		0.837		0.858
Change in post-dialysis MAP at 12 months	0.709	0.715		0.753		0.769
Change in antihypertensive medications at 3 months	0.147	0.131				
Change in antihypertensive medications at 6 months	0.187	0.166				
Change in antihypertensive medications at 9 months	0.283	0.267				
Change in antihypertensive medications at 12 months	0.313	0.308				
Cronbach’s alpha	0.812	0.817	0.663	0.872	0.671	0.868
Dillon-Goldstein's rho	0.836	0.850	0.854	0.894	0.857	0.899
Dijkstra-Henseler's rho	0.870	0.868	0.685	0.915	0.695	0.896
Average variance extracted	0.259	0.324	0.746	0.519	0.750	0.600
Heterotrait-monotrait ratio	<0.154	<0.227	<0.606		<0.681	

Note: MAP, mean arterial pressure

Table S2 - Evolution of the initial latent construct of “Fluid Volume” into the final latent constructs of “Fluid Volume” and “IDWG” for the measurement model

	Fluid Volume	Fluid Volume	IDWG	Fluid Volume	IDWG	Fluid Volume	IDWG	Fluid Volume	IDWG
	Initial after optimization of the “BP” latent construct	After separation of IDWG into a separate construct	After separation of IDWG into a separate construct	After removal 3 month indicators from BNP and NT-proBNP	After removal 3 month indicators from BNP and NT-proBNP	After removal of ECF/TBW ratio	After removal of ECF/TBW ratio	After removal 3 and 6 month indicators from OHI	After removal 3 and 6 month indicators from OHI
Change in LVEDV at 12 months	0.679	0.673		0.686		0.697		0.699	
Change in serum NT-Pro-BNP at 3 months	0.26	0.258							
Change in serum NT-Pro-BNP at 6 months	0.718	0.718		0.721		0.732		0.735	
Change in serum NT-Pro-BNP at 9 months	0.822	0.822		0.821		0.836		0.846	
Change in serum NT-Pro-BNP at 12 months	0.814	0.816		0.810		0.821		0.839	
Change in serum BNP at 3 months	0.481	0.477							
Change in serum BNP at 6 months	0.696	0.696		0.696		0.710		0.723	
Change in serum BNP at 9 months	0.825	0.829		0.828		0.846		0.865	
Change in serum BNP at 12 months	0.853	0.851		0.852		0.869		0.897	
Change in OHI at 3 months	0.506	0.515		0.504		0.456			
Change in OHI at 6 months	0.521	0.537		0.528		0.481			
Change in OHI at 9 months	0.6	0.601		0.596		0.577		0.567	
Change in OHI at 12 months	0.616	0.617		0.616		0.593		0.567	
Change in ECV/TBW at 3 months	0.454	0.467		0.446					
Change in ECV/TBW at 6 months	0.404	0.418		0.393					
Change in ECV/TBW at 9 months	0.478	0.484		0.463					
Change in ECV/TBW at 12 months	0.337	0.345		0.315					
Change in IDWG at 3 months	-0.201		0.728		0.713		0.713		0.713
Change in IDWG at 6 months	-0.183		0.814		0.811		0.811		0.807
Change in IDWG at 9 months	-0.085		0.839		0.847		0.847		0.847
Change in IDWG at 12 months	-0.073		0.797		0.804		0.804		0.804
Cronbach’s alpha	0.860	0.901	0.806	0.902	0.807	0.896	0.807	0.905	0.805

Dillon-Goldstein's rho	0.863	0.907	0.873	0.907	0.873	0.913	0.873	0.922	0.872
Dijkstra-Henseler's rho	0.946	0.948	0.816	0.944	0.824	0.937	0.824	0.939	0.824
Average variance extracted	0.298	0.385	0.633	0.410	0.633	0.500	0.633	0.534	0.630
Heterotrait-monotrait ratio	<0.692	<0.694		<0.716		<0.704		<0.693	

Note: LVEDV, left ventricular end diastolic volume; BNP, B-type natriuretic peptide; NT-pro-BNP, N-terminal-pro-BNP; OHI, overhydration index; ECV/TBW, extra-cellular fluid volume/total body water; IDWG, inter-dialytic weight gain

Table S3 - Finite mixture partial least squares (FIMIX-PLS) comparison for the structural model

Groups	1	2	3
AIC	1601.701	1494.308	1471.426
AIC3	1620.701	1533.308	1530.426
AIC4	1639.701	1572.308	1589.426
BIC	1651.008	1595.517	1624.538
CAIC	1670.008	1634.517	1683.538
HQ	1621.651	1535.257	1533.375
MDL5	2000.237	2312.356	2708.986
LnL	-781.85	-708.154	-676.713
EN		0.802	0.777
NFI		0.851	0.805
NEC		0.185	0.231

Note: AIC, Akaike's Information Criterion; AIC3, Modified AIC with Factor 3; AIC4, Modified AIC with Factor 4, ; BIC, Bayesian Information Criteria, CAIC, Consistent AIC; HQ, Hannan Quinn Criterion; MDL5, Minimum Description Length with Factor 5; LnL, Log Likelihood; EN, Entropy Statistic (Normed); NFI, Non-Fuzzy Index; NEC, Normalized Entropy Criterion

Table S4 – PLS-GAS comparison for the structural model

	Total	Group 1	Group 2
Observations	99	50	49
Goodness of Fit	0.303	0.301	0.330
Loadings			
Change in LVMI at 12 months	1	1	1
Dialysate [Na^+] allocation	1	1	1
Change in home dialysis MAP at 6 months	0.859	0.89	0.812
Change in home dialysis MAP at 12 months	0.871	0.89	0.872
Change in pre-dialysis MAP at 6 months	0.699	0.723	0.689
Change in pre-dialysis MAP at 9 months	0.822	0.844	0.818
Change in pre-dialysis MAP at 12 months	0.678	0.734	0.647
Change in post-dialysis MAP at 6 months	0.749	0.828	0.614
Change in post-dialysis MAP at 9 months	0.875	0.898	0.833
Change in post-dialysis MAP at 12 months	0.764	0.775	0.737
Change in antihypertensive medications at 3 months	0.843	-0.097	0.782
Change in antihypertensive medications at 6 months	0.948	-0.398	0.939
Change in antihypertensive medications at 9 months	0.911	-0.714	0.901
Change in antihypertensive medications at 12 months	0.685	-0.788	0.54
Change in LVEDV at 12 months	0.683	0.503	0.746
Change in serum NT-pro-BNP at 6 months	0.74	0.851	0.721
Change in serum NT-pro-BNP at 9 months	0.845	0.828	0.851
Change in serum NT-pro-BNP at 12 months	0.837	0.795	0.882
Change in serum BNP at 6 months	0.719	0.54	0.748
Change in serum BNP at 9 months	0.853	0.865	0.863
Change in serum BNP at 12 months	0.881	0.782	0.911
Change in OHI at 6 months	0.462	0.562	0.457
Change in OHI at 9 months	0.58	0.444	0.631
Change in OHI at 12 months	0.595	0.587	0.612
Change in IDWG at 3 months	0.711	0.774	0.666
Change in IDWG at 6 months	0.81	0.703	0.864
Change in IDWG at 9 months	0.845	0.808	0.867
Change in IDWG at 12 months	0.806	0.788	0.825
Path coefficients			
Inter-dialytic BP -> LVMI	0.219	0.258	0.166
Dialytic BP -> LVMI	0.23	0.192	0.251
Fluid Volume -> LVMI	0.509	0.407	0.569
IDWG -> LVMI	-0.055	-0.075	-0.064
Dialysate [Na^+] allocation -> Inter-dialytic BP	-0.081	-0.076	-0.05
Antihypertensive medications -> Inter-dialytic BP	-0.055	-0.114	-0.065
Fluid Volume -> Inter-dialytic BP	0.215	0.317	0.197
Dialysate [Na^+] allocation -> Dialytic BP	-0.095	-0.168	0.042
Antihypertensive medications -> Dialytic BP	0.005	-0.137	0.028
Fluid Volume -> Dialytic BP	0.323	0.384	0.357
Dialysate [Na^+] allocation -> Antihypertensive medications	-0.195	-0.049	-0.428
Dialysate [Na^+] allocation -> Fluid Volume	-0.069	-0.187	0.011
Dialysate [Na^+] allocation -> IDWG	-0.368	-0.399	-0.346

Note: LVMI, left ventricular end mass index; MAP, mean arterial pressure; LVEDV, left ventricular end diastolic volume; BNP, B-type natriuretic peptide; NT-pro-BNP, N-terminal-pro-BNP; OHI, overhydration index; ECV/TBW, extra-cellular fluid volume/total body water; IDWG, inter-dialytic weight gain

Supplementary Figures

Figure S1a. Structural model in GAS 1 subgroup

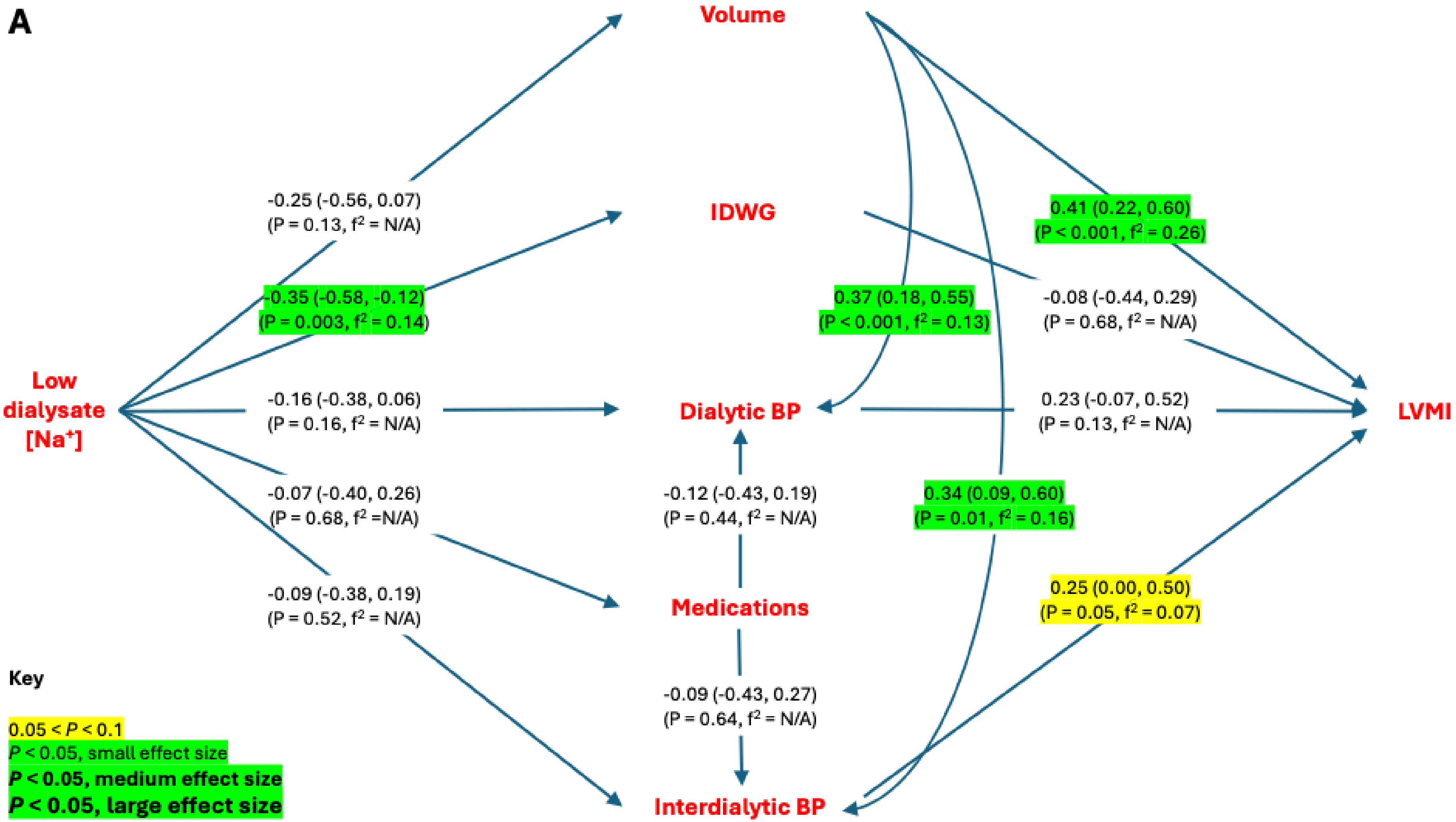

Figure S1b. Structural model in GAS 2 subgroup

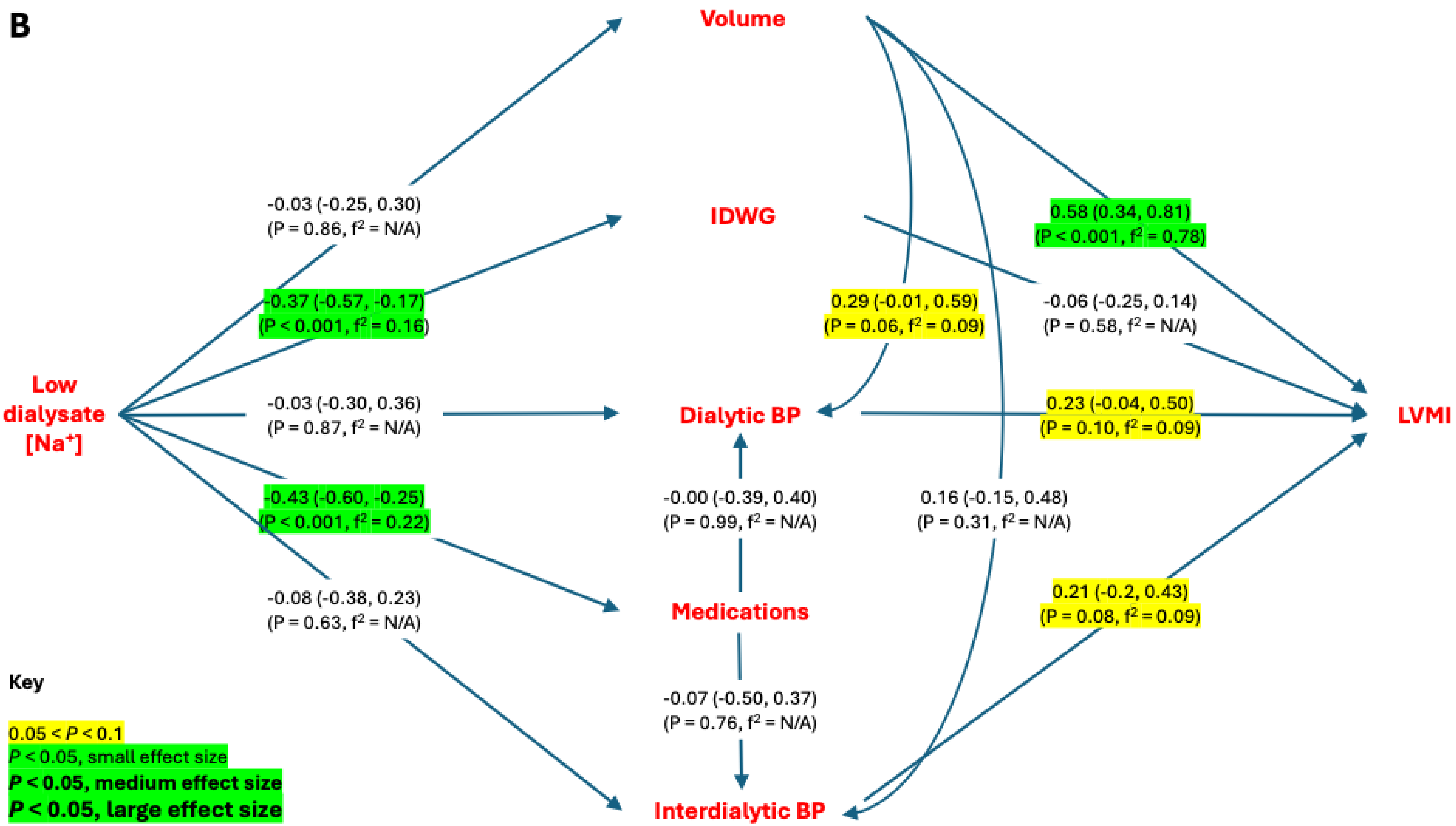

Item S1. Detailed Statistical Methods

Introduction to various approaches to structural equation modelling (SEM)

Structural equation modelling (SEM) is a statistical technique used to analyse complex relationships between observed and latent variables, using two sets of equations, solved simultaneously[1]. The first set enables researchers to identify latent variables (for example in this study, “volume overload”), which are constructed at an observed level from measured indicator variables (e.g.in this case, extracellular fluid volume (ECV), B-type natriuretic peptide (BNP) etc). This set of equations is collectively referred to as the outer or measurement model. The second set allows the evaluation of relationships among these latent constructs at a theoretical level (continuing this example, “what is the effect of dialysate [Na+] on volume status and simultaneous effect of volume status on left ventricular mass index (LVMI)”). This second set of equations is referred to as the inner, structural, or path model.

Path analysis is the oldest explicit statistical technique of causal analysis, as developed by geneticist Sewall Wright in the 1920s-1930s[3,4]. Current forms of SEM developed in the 1960s when researchers combined path analysis with factor analysis from psychometrics and simultaneous equation modelling from econometrics[5]. Since that time, SEMs have been the mainstay of causal analysis in the social and behavioural sciences [5-9]. The technique is now commonplace in other areas such as the environmental sciences[10-17], and is being increasingly used in the health sciences[18-23]. SEM is an appropriate approach for causal modelling when[24-28]:

- There is strong theoretical justification for a causal structure which are input into the model, rather than being discovered by the analysis
- The research design supports causal inference (experimental, longitudinal with temporal precedence, or some other appropriate quasi-experimental design), with the acknowledgement that cross-sectional SEM alone cannot establish causality

- Issues around identification and confounding are addressed through correct model specification.

Ultimately, SEM tests whether empirical data are consistent with a causal hypothesis, rather than proving whether the hypothesis is true.

Classical structural equation modelling is different from standard regression in several fundamental ways. Regression models are computed so that parameters predict values for each person (or case) that are as close as possible to observed ones. For example, in linear regression this is achieved by minimizing (Eq 1):

$$\Sigma(y_i - \hat{y}_i)^2 = \Sigma(y_i - \beta_0 - \beta_1 x_i)^2 \quad \text{Eq 1}$$

Building a regression model involves the following broad steps:

- **Input:** Individual data points (n × k matrix of raw data)
- **Process:** Fit a function through the points
- **Output:** Coefficients that minimize individual prediction errors

In contrast, classical SEMs are computed so that parameters make the model-implied covariances match the observed covariances, by minimizing (Eq 2):

$$F(\Sigma, S) = \log|\Sigma| + tr(S\Sigma^{-1}) - \log|S| - p \quad \text{Eq 2}$$

Where:

S = observed covariance matrix (from raw data)

Σ = model-implied covariance matrix (from estimated parameters in the SEM)

p = number of variables

log|·| = natural log of the determinant

tr(·) = trace (sum of diagonal elements)

Building a classical SEM involves the following broad steps:

- **Input:** Covariance matrix (k × k summary of relationships)
- **Process:** Construct a model that would generate these covariances

- **Output:** Multiple parameter matrices that reproduce the covariance structure

An alternative computational approach to covariance-based SEM (CB-SEM)[29] is through the use of variance-based SEM, most commonly referred to as partial least squares SEM (PLS-SEM)[30-36]. PLS-SEM handles measurement models, multiple equations, indirect effects, and structural relationships, and can be regarded as a purely regression-based approach to SEM. The classical CB-SEM approach is based on the common factor model, incorporating indicator variables into latent constructs using factor analysis, and confirming theories by evaluating how accurately the proposed structural model reproduces the observed covariance matrix for the given data. In contrast, the PLS-SEM procedure incorporates indicator variables into latent constructs as weighted composites from a linear combination of observed indicators, using multiple regression analysis to estimate path coefficients in the structural model that maximise explained variance of the model's dependent variable(s).

Within each method, there are subtle differences between computations in measurement models when they are reflective (that is, when the indicators are all manifestations of the latent construct) versus those that are formative (that is, when the indicators are all different facets that when combined form the latent construct). In CB-SEM, reflective latent constructs are derived from common factor models, whereas formative ones are derived as weighted linear composites. These are not "true" linear composites (that is, where the latent variable is totally determined by its indicators, with no causes beyond them - technically speaking, disturbance = 0) but rather ones estimated to be consistent with an empirically derived latent variable defined by the covariance structure of its causal indicators (that is, there is potential for other missing or unmeasured causes and indicators – technically speaking, disturbance ≠ 0). In PLS-SEM, the approach to reflective measurement models (a.k.a. Mode A) is shown in Box 1 below, and the approach to formative ones (a.k.a. Mode B) in Box 2. Both approaches generate true composites for latent constructs (disturbance = 0), albeit within different algorithms.

There is ongoing controversy about whether PLS-SEM and CB-SEM provide equivalent statistical and substantive numerical estimates. For computations involving the measurement models, it is argued that PLS-SEM is less appropriate (and therefore less

accurate) for reflective ones[37], and that CB-SEM is less appropriate for formative ones[38,39]. These arguments are based largely in theory, from the way that computations are made in respective approaches described above. Estimates from PLS-SEM are weighted composites that are inherently formative[37]. Those from CB-SEM are factor-based and largely synonymous with reflective measurement. The methods to generate composites for formative measurement in CB-SEM are literally “retrofit” and potentially measure an incomplete version of the ontologically real construct[38,40,41]. The weight of most recent opinion, however, is that both approaches present mathematical representations of focal concepts and their associated definitions, and any measurement be it factor-based or composite-based simply approximates the true underlying concept[42].

There are numerical sources of bias specific to PLS-SEM that arise during computations. First, loadings in the measurement model are inconsistent - meaning they do not converge to true population values even with infinite sample size (i.e. estimates of factor loadings never converge to the true value)[43-47]. This is inherent in the use of composite rather than factor scores, where the PLS algorithm creates weights in the presence of measurement error. These estimates converge to values proportional to true factors, but not ones that are equal to them (see Box 1). This contrasts with CB-SEM where the approach estimates loadings that converge to the true values with correct accounting for measurement error. Without going through the covariance algebra, the indicator loading in PLS-SEM will be approximately:

	$w_i \approx \sqrt{\rho} \times \lambda$	Eq 3

Where:

w = PLS weights
ρ = construct reliability
λ = true factor loadings

The generation of the composite from loadings in the PLS-SEM algorithm is therefore biased downwards. Practically, however, this bias is often offset in empirical studies since the iterative PLS algorithms are also affected by the systematically higher correlations between

indicators and composite scores in the presence of measurement error, which biases them upwards. In summary, therefore, PLS-SEM indicator loadings are systematically inconsistent compared to those with CB-SEM since the former methodology does not correctly account for measurement error while the latter does. These error-contaminated composites lead to further numerical bias since they are used to estimate structural paths between constructs. This second source of bias is an attenuation one whereby path coefficients are typically underestimated. These issues can be overcome with the use of a correction factor that has been recently published[44]. This correction is based on construct reliability (ρ) which is then applied to indicator loadings thereby correcting attenuation bias in path coefficients (Eq 4). This results in structural model estimates that converge to true population parameters as sample size increases:

$$\lambda\_PLSc = \lambda\_PLS / \sqrt{\rho} \quad \text{Eq 4}$$

A key limitation of this approach is that it only works with reflective measurement models and not formative ones, because the correction relies on assumptions about measurement error that do not apply in the latter situation.

For all these potential limitations, PLS-SEM has advantages over CB-SEM that outweigh disadvantages[48,49]. First, the approach is unquestionably superior to CB-SEM with non-normal data, where it does not tend to underestimate standard errors so much or inflate goodness-of-fit indices[50]. Secondly, PLS-SEM achieves comparatively better convergence than CB-SEM when the sample size is small, thus being effective for research with relatively small sample sizes such as this one[50]. Finally, PLS-SEM does not encounter the convergence problems that are so common with CB-SEM - PLS-SEM almost always converge due to the absence of likelihood functions, a lack of identification constraints, and a mathematically simple and robust algorithm that only rarely gets into computational problems.

Empirically, the use of PLS-SEM in the social science and marketing literature has provided insights that are useful with high face validity. Several published articles (including one by the main author) have directly compared CB-SEM and PLS-SEM estimates for the same structural models. The results of these replication studies were as expected given the

methodical differences described above, although both methods provide directionally similar estimates across measurement and structural models, with only minor differences in terms of effect size [51-59]: Comparing the estimates between CB-SEM and PLS-SEM, the following insights arise:

- Measurement model parameters (loadings, average variance explained, Cronbach's alpha) tended to be slightly higher in PLS-SEM using reflective measurement models, and higher in CB-SEM when using formative models.
- Structural path coefficients tended to be slightly stronger in CB-SEM, and weaker with PLS-SEM

Technical differences aside, it is widely accepted that PLS-SEM is the most appropriate method for prediction and theory development, while classical CB-SEM remains the standard for theory testing and confirmation.

As with CB-SEM, several robust guidelines exist for the conduct and reporting of PLS-SEM analyses that help with consistent interpretation of the reported studies in the literature[33,49,50,53,60,61].

Overall approach to structural equation modelling in this study

We used PLS-SEM for this study, motivated by common finding of non-normality in survey data, the small sample size of our study, and our need to explore the structure of latent constructs, test research hypotheses, and assess multiple causal relationships.

We used a mixture of reflective and formative measures for predicting the main outcome in the study, with the formative ones being the main predictor (lower dialysate [Na^+] allocation) and the main outcomes (LV mass index). We did not use consistent PLS-SEM because of the presence of formative constructs in the model.

We used an open-source implementation of the PLS-SEM methodology[34,62] in Stata 18 software.

Box 1: Simplified steps for reflective measurement models in PLS-SEM ("Mode A"):

- Arrows point **from** construct **to** indicators, and all indicators arise from a common causal construct and are highly intercorrelated (for example, anhedonia and anorexia as correlated indicators of depression)
- Consider latent construct ξ_j estimated within a path model, and indicated by X_1, X_2, X_3 in the corresponding measurement model
- PLS alternates cyclically between the following two steps until convergence of weights calculated between Steps 1 and 2, starting with arbitrary values for initial weights in the model

Step 1:

- The weighted relationship between each indicator and ξ is modelled as a simple correlation between indicators in the outer model and the current estimate of ξ_j from the inner model as determined in Step 2 (Eq A):

	$w_i = cor(\xi\_j, X_i)$	Eq A

- With standardized data (typical in PLS-SEM), this is equivalent to (Eq B):

	$w_i = cov(\xi\_j, X_i)$	Eq B

- The updated composite $\hat{y}$ that defines the new value for ξ_j for the next iteration is then calculated by the sum of weighted indicators (Eq C):

	$\hat{y} = w_1 \cdot X_1 + w_2 \cdot X_2 + w_3 \cdot X_3$	Eq C

Step 2 (using path scheme):

- An updated value for ξ_j for latent is estimated in the path model using the structural relationships of current value from $\hat{y}$ in Step 1 with the other adjacent constructs
- For endogenous constructs (has incoming paths), the new estimate for ξ_j is based on its predecessor constructs k: (Eq D):

$$\xi_j = \Sigma\, e_{jk} \times \xi_k \quad \text{Eq D}$$

Where the inner weights e_{jk} are the multiple regression coefficients of ξ_j on all its predecessors (Eqs E and F):

$$\xi_j = \beta_1 \cdot \xi_{k1} + \beta_2 \cdot \xi_{k2} + \ldots + \beta_p \cdot \xi_{kp} + \varepsilon \quad \text{Eq E}$$

$$e_{jk} = \beta_k \quad \text{Eq F}$$

- For exogenous constructs (only outgoing paths, no incoming), the estimate for ξ_j is based on its successor constructs k: (Eq G):

$$\xi_j = \Sigma\, e_{jk} \times \xi_k \quad \text{Eq G}$$

Inner weights e_{jk} are calculated as the simple correlation with each successor (Eq H):

$$e_{jk} = cor(\xi_j, \xi_k) \quad \text{Eq H}$$

- The algorithm returns to Step 1 for next cycle, repeating for cyclically until inner and outer calculations of weights converge (typically when changes in weights < 0.00001)

Box 2: Simplified steps for formative measurement models in PLS-SEM ("Mode B"):

- Arrows point **from** indicators **to** constructs, where indicators cause the common construct (i.e. dropping an indicator can change the construct's meaning), and are not necessarily correlated (for example, trust in the doctor and easy parking are separate and non-interchangeable indicators of patient satisfaction and may vary independently)
- Consider latent construct ξ estimated within a path model, and composed of indicators X_1, X_2, X_3 in the corresponding measurement model
- PLS alternates cyclically between the following two steps until convergence of weights calculated between Steps 1 and 2, starting with arbitrary values for initial weights in the model

Step 1:

- The weighted relationship between each formative indicator and ξ is determined by multiple regression indicators in the outer model and the current estimate of ξ_j from the inner model as determined in Step 2 (Eq A):

	$\xi_j = \beta_1 \cdot X_1 + \beta_2 \cdot X_2 + \beta_3 \cdot X_3 + \varepsilon$	Eq A

- The outer weights are set to the regression coefficients: (Eq B):

	$w_i = \beta_i$	Eq B

- The updated composite ŷ that defines the new value for ξ_j for the next iteration is then calculated by the sum of weighted indicators (Eq C):

	$\hat{y} = w_1 \cdot X_1 + w_2 \cdot X_2 + w_3 \cdot X_3$	Eq C

Step 2 (using path scheme) is identical to that in "Mode A"

Detailed methods for building the measurement model in this study

In developing the measurement model, we started with defining latent variables that had previously been well established as being clinically relevant to LVMI, namely "volume status" and "blood pressure"[60-62], acknowledging the wide range of afterload / preload-independent factors also considered as important[63-67]. The indicator variables for these initial latent constructs were selected from the secondary outcomes recorded in the SoLID trial. To ensure content validity of these constructs, the indicator variables were initially selected to fit the conceptual definition of the latent variable, based on previous publications by this group[68] and the other academic experts in the therapeutic area cited above.

In refining the measurement model[33,34,39,49], we assessed the validity of constructs before finalizing model. This was done carefully because of the exploratory rather than confirmatory nature of the research, the lack of theory upon which to specify the model, and the small body of literature reporting simultaneous outcomes along the causal pathway between the study intervention and outcome in the target population. We were therefore keen to ensure that there was a consistent conceptual definition of the constructs, and that the indicators that were clear and consistent manifestations of their respective constructs with high face validity. Importantly, this exercise was not done to introduce theoretical ambiguity, mask causal direction, or weaken construct definition - PLS-SEM almost always converge for reasons previous explained, without the need to introduce empirical convenience in this way.

We first examined indicator loadings of measures (indicator reliability), where the default threshold level is 0.7 or above, ensuring that the latent construct explains ~50% of the variance in the indicator [76]. We reduced this threshold to 0.6 due to this exercise being a frankly exploratory one [33], and we retained all indicators with loadings 0.6 and above automatically. Indicators with very low loadings (below 0.40) were removed if there was a strong theoretical reason to do so. Indicators with a loading between 0.4 and 0.6 were considered for removal but only if this improved internal consistency or convergent validity to well beyond recommended thresholds. We erred towards retaining them if the average

variance explained was 0.5 or above and the overall composite reliability looked acceptable[33,49,77,78].

Next, we examined for unidimensionality. This was done to ensure that the model had the fewest number of constructs to explain the greatest amount of variance in the indicators. Principal component analysis was performed for each construct, and the resulting eigenvalues (the amount of variance a construct explains in a set of indicators) examined. Consistent with guidelines, we aimed to retain constructs with one component and one component only with an eigenvalue above (or well above) 1.

Third, we assessed the internal consistency of the latent variables (changes in one indicator mean changes in others). This was done through Cronbach's alpha, Dillon-Goldstein's rho (DG rho), and Dijkstra-Henseler's rho (rho_A), where values between 0.70 and 0.93 are recommended for Cronbach's alpha and DG rho, while above 0.70 is recommended for rho_A[72] [38,46,57].

Fourth, we assessed construct validity, defined as the extent to which the different indicators reflect the latent variable, as assessed from convergent and discriminant validity[32,33]. We assessed convergent validity from how well the latent constructs converged to predict the variance of its indicators, calculating the average variance extracted with a recommended value of above 0.50. We assessed discriminant validity as the degree to which a given latent construct differed from the others, using the heterotrait-monotrait ratio[73], which should be below 0.90.

Depending on these all these results, the initial latent constructs were changed or expanded upon or new ones added, and the indicator variable selection refined. The criteria for selecting a final model included high face validity in a clinical sense, high correlation between indicators and their respective reflective latent variable, no correlated errors, invariance across groups and time, and no direct effects with other indicators.

Detailed methods for building the structural models in this study

After ensuring that the constructs were reliable and valid, we then developed the structural models for the main outcome, namely LV mass index. In view of the small simple size, no moderating variables were considered in the main model. We used simple correlational analysis to identify the key scales associating either positively or negatively with the outcome of interest. We then examined matrices of coefficients in an initial SEM specifying every possible causal relationship between all the refined constructs with the outcome, clinically plausible or not. We then placed the latent constructs into a causal pathway, with plausible structural relationships derived from both the literature and our own cumulative clinical experience.

We then assessed the structural model for robustness. This included the use of standard evaluation criteria such as construct-specific coefficient of determination / explanatory power (R^2) and the predictive accuracy/blindfolding-based cross-validated redundancy measure (Q^2)[33,34,39,81]. PLS-SEM does not have well-accepted criteria for goodness-of-model-fit. Using the average R^2, the following interpretations are suggested: $R^2 < 0.1$ is unacceptable in the social sciences but often acceptable in health; up to 0.25 indicates acceptable but mediocre fit in most settings; up to 0.67 indicates good fit; > 0.67 indicates superior fit [31,82-85]. Using the Q^2, values above 0 indicate some predictive relevance, and values above 0.35 indicate strong relevance. Model fit was also assessed by the standardised root mean square residual (SRMR), using a value below the threshold value of 0.08 in the saturated and the estimated model as being indicative of excellent model fit[60]. The structural model was evaluated for collinearity following the guidelines in the literature (the variance inflation factor, VIF) with recommended values below 2.5.

After assessing the models for robustness, hypotheses testing was conducted with 10,000 bootstrap samples, determined at a significance level of 0.05.

We report standardized parameter estimates, interpreted as the number of standard deviations by which the outcome variable mean increases for every 1 standard deviation increase of the predictor variable. In this study, we defined effect sizes by β values and Cohen's f^2: β values of 0.05, 0.1 and 0.25 represent respectively a small, medium and large effect, and Cohen's f^2 values of 0.02, 0.15 and 0.35 represent respectively a small, medium and large effect[79].

Moderation by baseline pre-dialysis serum [Na^+] was investigated through subgroup analysis and group-specific path modelling according to this potential moderator, comparing estimates between models, in terms of the effect of the main predictor on the outcome, and also the estimates between intermediate outcomes on the causal / structural pathway.

Unobserved heterogeneity was assessed by exploring whether there were further hidden sub-groups that differed significantly from each other in the model[80]. We used finite mixture partial least squares (FIMIX-PLS) in the first instance to search for homogeneous groups, which it does by identifying groups within data that share the same characteristics in terms of inner model weights, and outputting an estimation of how likely each observation belongs to one of the groups. We then applied PLS using genetic algorithm segmentation to more closely examine indicator loadings and structural coefficients between identified groups. The number of groups tested by these techniques is user specified, and we applied an initial input using a “rule of thumb” based on the number of structural determinants of LVMI and the overall sample size. Group-specific path modelling was undertaken for outcomes, thus allowing further differentiated and more precise conclusions to be formed.

Missing data were imputed using the k-th nearest neighbour method.

Supplementary References

1. Beran TN, Violato C. Structural equation modeling in medical research: a primer. *BMC Research Notes*. 2010/10/22 2010;3(1):267. doi:10.1186/1756-0500-3-267

2. Wright S. The Method of Path Coefficients. *The Annals of Mathematical Statistics*. 1934;5(3):161-215.

3. Wright S. Correlation and causation. *Jour Ag Res* 1921;20:557-585.

4. Pearl J. Graphs, Causality, and Structural Equation Models. *Sociological Methods & Research*. 1998;27(2):226-284. doi:10.1177/0049124198027002004

5. Bentler PM. Causal Modeling via Structural Equation Systems. In: Nesselroade JR, Cattell RB, eds. *Handbook of Multivariate Experimental Psychology*. Springer US; 1988:317-335.

6. Pearl J. *Causality: Models, Reasoning, and Inference*. Cambridge University Press; 2000.

7. Pearl J. An introduction to causal inference. *Int J Biostat*. Feb 26 2010;6(2):Article 7. doi:10.2202/1557-4679.1203

8. Bollen KA. *Structural equations with latent variables*. Wiley series in probability and mathematical statistics. John Wiley and Sons; 1989.

9. Pearse IS, Becker Z, Ode PJ, Gaskin JF, West NM. Low genetic diversity in populations of a clonal invasive plant limits sexual reproduction. *Am J Bot*. Aug 2025;112(8):e70083. doi:10.1002/ajb2.70083

10. Mancin E, Sartori C, Rulli E, et al. Indicators of resilience in cattle based on test-day milk yield: comparison of within- and between-lactation and their relationship with milk production. *Animal*. Sep 2025;19(9):101596. doi:10.1016/j.animal.2025.101596

11. Villacañas de Castro C, Musculus J, Timmermann E, Lühken R, Kiel E, Sauer FG. Carry-over effects in Culex species along a land use gradient with differences in microclimatic conditions. *Parasit Vectors*. Jul 4 2025;18(1):256. doi:10.1186/s13071-025-06903-y

12. Yu B, Lu K, Liu D, Nie L, Bian Z, Gao H. Exploring microbial drivers of DOM removals and greenhouse gas emissions in a centralized wastewater treatment plant of a large-scale coal industrial park. *Environ Res*. Dec 1 2025;286(Pt 3):122981. doi:10.1016/j.envres.2025.122981

13. Celegeer, Watanabe N, Otani K, Okada T, Matsushita M, Nakagawa M. Factors affecting seedling establishment in a warm-temperate secondary forest affected by oak wilt disease. *Plant Biol (Stuttg)*. Oct 2025;27(6):1097-1106. doi:10.1111/plb.70051

14. Fukasawa Y, Kimura S, Kominami Y, et al. Oak Wilt Disease May Reduce the Initial Decay Rate of Dead Quercus serrata Stems by Altering Fungal Communities in the Wood. *Environ Microbiol*. Jan 2025;27(1):e70026. doi:10.1111/1462-2920.70026

15. Ao G, Wang C, Yang L, et al. Quorum sensing: the "switch" in the competitive relationship between Gram-positive bacteria based on transcriptomic analysis. *Microbiol Res*. Jan 2025;290:127961. doi:10.1016/j.micres.2024.127961

16. Li Z, Chai Y, Gao C, Li J, Cui K. The heterogeneity of Pinus yunnanensis plantation growth was driven by soil microbial characteristics in different slope aspects. *BMC Plant Biol*. Jan 27 2025;25(1):114. doi:10.1186/s12870-025-06152-2

17. Chalwe JM, Grobler C, Oldewage-Theron W. Development of a Structural Equation Model to Examine the Relationships between Genetic Polymorphisms and Cardiovascular Risk Factors. *Nutrients*. May 25 2023;15(11)doi:10.3390/nu15112470

18. Wu L, Chen K, Zhang Z, et al. The Relationship Between Glymphatic Function, White Matter Hyperintensity and Cognition: A Structural Equation Model MRI Study. *CNS Neurosci Ther*. Jun 2025;31(6):e70478. doi:10.1111/cns.70478
19. Yao Y, Shao H, Masters J, et al. Cardiac surgery with valve replacement temporarily disrupts the hippocampal memory network. *Br J Anaesth*. Feb 2025;134(2):402-413. doi:10.1016/j.bja.2024.10.022
20. Tong M, Li Y, Rong K, Sun Q, Dai J, Huang Y. Structural equation model analysis of the effect of visceral fat on osteoporosis. *J Orthop Surg Res*. Jul 16 2024;19(1):408. doi:10.1186/s13018-024-04888-5
21. Lv Z, Yuan L, Mao Y, Ai S. Recurrent laryngeal nerve paralysis as a potential mediator of complications in esophagectomy following lymph node dissection. *Dis Esophagus*. May 3 2025;38(3)doi:10.1093/dote/doaf028
22. Houdmont M, Lim EH, Djohan Hartanto A, et al. Pathological Q waves at presentation of anterior ST segment elevation myocardial infarction predict heart failure: a Southeast Asian perspective. *Coron Artery Dis*. Aug 1 2025;36(5):378-383. doi:10.1097/mca.0000000000001475
23. Bollen KA. *Structural equations with latent variables*. Structural equations with latent variables. John Wiley & Sons; 1989:xiv, 514-xiv, 514.
24. Pearl J. *Causality*. 2 ed. Cambridge University Press; 2009.
25. Bollen K, Pearl J. Eight Myths About Causality and Structural Equation Models. 2013:301-328.
26. MacCallum RC, Austin JT. Applications of structural equation modeling in psychological research. *Annu Rev Psychol*. 2000;51:201-26. doi:10.1146/annurev.psych.51.1.201
27. Kline RB. *Principles and practice of structural equation modeling, 5th ed*. The Guilford Press; 2023:494.
28. Jöreskog KG. A General Approach to Confirmatory Maximum Likelihood Factor Analysis. *Psychometrika*. 1969;34(2):183-202. doi:10.1007/BF02289343
29. Wold H. Path Models with Latent Variables: The NIPALS (Nonlinear Iterative PArtial Least Squares) approach. In: Blalock HM, Aganbegian A, Borodkin FM, Boudon R, Capecchi V, eds. *Quantitative Sociology*. Academic Press; 1975:307-357.
30. Hair JF, Ringle CM, Sarstedt M. PLS-SEM: Indeed a Silver Bullet. *Journal of Marketing Theory and Practice*. 2011/04/01 2011;19(2):139-152. doi:10.2753/MTP1069-6679190202
31. Hair JF, Sarstedt M, Ringle CM, Mena JA. An assessment of the use of partial least squares structural equation modeling in marketing research. *Journal of the Academy of Marketing Science*. 2012/05/01 2012;40(3):414-433. doi:10.1007/s11747-011-0261-6

32. Hair JF, Hult GTM, Ringle CM, Sarstedt M. *A Primer on Partial Least Squares Structural Equation Modeling (PLS-SEM)*. 3rd ed. Sage Publications Inc.; 2022.

33. Mehmetoglu M, Venturini S. *Structural Equation Modelling with Partial Least Squares Using Stata and R*. 1st ed. CRC Press; 2021.

34. Wold H. Model Construction and Evaluation When Theoretical Knowledge Is Scarce. *Evaluation of Econometric Models*. National Bureau of Economic Research, Inc; 1980:47-74.

35. Graff J, Schmidt P. A general model for decomposition of effects. *Systems Under Indirect Observation: Causality, Structure, Prediction (K Jöreskog and H Wold, eds) North-Holland, Amsterdam*. 1982:131-148.

36. Henseler J, Schuberth F, Lee N, Kemény I. Why researchers should be cautious about using PLS-SEM. *Industrial Marketing Management*. 2025/07/01/ 2025;128:A8-A15. doi:https://doi.org/10.1016/j.indmarman.2024.01.017

37. Jarvis CB, MacKenzie SB, Podsakoff PM. A Critical Review of Construct Indicators and Measurement Model Misspecification in Marketing and Consumer Research. *Journal of Consumer Research*. 2003;30(2):199-218. doi:10.1086/376806

38. Sarstedt M, Hair JF, Pick M, Liengaard BD, Radomir L, Ringle CM. Progress in partial least squares structural equation modeling use in marketing research in the last decade. *Psychology & Marketing*. 2022/05/01 2022;39(5):1035-1064. doi:https://doi.org/10.1002/mar.21640

39. Guenther P, Guenther M, Ringle CM, Zaefarian G, Cartwright S. PLS-SEM and reflective constructs: A response to recent criticism and a constructive path forward. *Industrial Marketing Management*. 2025/07/01/ 2025;128:1-9. doi:https://doi.org/10.1016/j.indmarman.2025.05.003

40. Dijkstra T. Some comments on maximum likelihood and partial least squares methods. *Journal of Econometrics*. 1983/05/01/ 1983;22(1):67-90. doi:https://doi.org/10.1016/0304-4076(83)90094-5

41. Dijkstra TK, Henseler J. Consistent Partial Least Squares Path Modeling. *Management Information Systems Quarterly*. 2015;39(2):297-316. doi:10.25300/misq/2015/39.2.02

42. Esposito Vinzi V, Chin W, Henseler J, Wang H. *Handbook of Partial Least Squares: Concepts, Methods and Applications*. 2010.

43. Rönkkö M, Evermann J. A Critical Examination of Common Beliefs About Partial Least Squares Path Modeling. *Organizational Research Methods*. 2013;16(3):425-448. doi:10.1177/1094428112474693

44. McIntosh CN, Edwards JR, Antonakis J. Reflections on Partial Least Squares Path Modeling. *Organizational Research Methods*. 2014;17(2):210-251. doi:10.1177/1094428114529165

45. Hair JF, Matthews LM, Matthews RL, Sarstedt M. PLS-SEM or CB-SEM: updated guidelines on which method to use. *International Journal of Multivariate Data Analysis*. 2017/01/01 2017;1(2):107-123. doi:10.1504/IJMDA.2017.087624

46. Hair JF, Risher JJ, Sarstedt M, Ringle CM. When to use and how to report the results of PLS-SEM. *European Business Review*. 2019;31(1):2-24. doi:10.1108/EBR-11-2018-0203

47. Hair JF, Sarstedt M, Hopkins L, Kuppelwieser VG. Partial least squares structural equation modeling (PLS-SEM). *European Business Review*. 2014;26(2):106-121. doi:10.1108/EBR-10-2013-0128

48. Dash G, Paul J. CB-SEM vs PLS-SEM methods for research in social sciences and technology forecasting. *Technological Forecasting and Social Change*. 2021/12/01/ 2021;173:121092. doi:https://doi.org/10.1016/j.techfore.2021.121092

49. Hair JF, Hult GTM, Ringle CM, Sarstedt M, Thiele KO. Mirror, mirror on the wall: A comparative evaluation of composite-based structural equation modeling methods. *Journal of the Academy of Marketing Science*. 2017;45(5):616-632. doi:10.1007/s11747-017-0517-x

50. Hair JF, Matthews L, Matthews R, Sarstedt M. PLS-SEM or CB-SEM: updated guidelines on which method to use. *International Journal of Multivariate Data Analysis*. 01/01 2017;1:107. doi:10.1504/IJMDA.2017.087624

51. Mohamad M, Afthanorhan A, Awang Z, Mohammad M. Comparison Between CB-SEM and PLS-SEM: Testing and Confirming the Maqasid Syariah Quality of Life Measurement Model. *The Journal of Social Sciences Research*. 2019;

52. Reinartz W, Haenlein M, Henseler J. An empirical comparison of the efficacy of covariance-based and variance-based SEM. *International Journal of Research in Marketing*. 2009;26(4):332-344. doi:10.1016/j.ijresmar.2009.08.001

53. Rigdon EE, Sarstedt M, Ringle CM. On Comparing Results from CB-SEM and PLS-SEM: Five Perspectives and Five Recommendations. *Marketing: ZFP – Journal of Research and Management*. 2017;39(3):4-16.

54. Sarstedt M, Hair JF, Ringle CM, Thiele KO, Gudergan SP. Estimation issues with PLS and CBSEM: Where the bias lies! *Journal of Business Research*. 2016/10/01/ 2016;69(10):3998-4010. doi:https://doi.org/10.1016/j.jbusres.2016.06.007

55. Vuković M. CB-SEM vs PLS-SEM comparison in estimating the predictors of investment intention. *Croatian operational research review*. 10/07 2024;15:131-144. doi:10.17535/crorr.2024.0011

56. Marshall MR, Curd S, Kennedy J, et al. Structural Equation Modelling to Identify Psychometric Determinants of Medication Adherence in a Survey of Kidney Dialysis Patients. *Patient Prefer Adherence*. 2024;18:855-878. doi:10.2147/ppa.S454248

57. Henseler J, Hubona G, Ray PA. Using PLS path modeling in new technology research: updated guidelines. *Industrial Management & Data Systems*. 2016;116(1):2-20. doi:10.1108/IMDS-09-2015-0382
58. Benitez J, Henseler J, Castillo A, Schuberth F. How to perform and report an impactful analysis using partial least squares: Guidelines for confirmatory and explanatory IS research. *Information & Management*. 2020/03/01/ 2020;57(2):103168. doi:https://doi.org/10.1016/j.im.2019.05.003
59. Venturini S, Mehmetoglu M. plssem: A Stata Package for Structural Equation Modeling with Partial Least Squares. *Journal of Statistical Software*. 03/26 2019;88(8):1 - 35. doi:10.18637/jss.v088.i08
60. de Simone G. Left ventricular geometry and hypotension in end-stage renal disease: a mechanical perspective. *J Am Soc Nephrol*. Oct 2003;14(10):2421-7. doi:10.1097/01.asn.0000088724.66957.fc
61. Harnett JD, Parfrey PS. Blood pressure control and regression of LV hypertrophy in dialysis patients. *Contrib Nephrol*. 1994;106:110-3. doi:10.1159/000422934
62. London GM, Marchais SJ, Guerin AP, Metivier F, Pannier B. Cardiac hypertrophy and arterial alterations in end-stage renal disease: hemodynamic factors. *Kidney Int Suppl*. Jun 1993;41:S42-9.
63. Glassock RJ, Pecoits-Filho R, Barberato SH. Left ventricular mass in chronic kidney disease and ESRD. *Clin J Am Soc Nephrol*. Dec 2009;4 Suppl 1:S79-91. doi:10.2215/cjn.04860709
64. Ritz E. Left ventricular hypertrophy in renal disease: beyond preload and afterload. *Kidney Int*. Apr 2009;75(8):771-3. doi:10.1038/ki.2009.35
65. Ritz E, Wanner C. The Challenge of Sudden Death in Dialysis Patients. *Clinical Journal of the American Society of Nephrology*. 2008;3(3)
66. Parker T, 3rd, Hakim R, Nissenson AR, Steinman T, Glassock RJ. Dialysis at a crossroads: 50 years later. *Clin J Am Soc Nephrol*. Feb 2011;6(2):457-61. doi:10.2215/cjn.08450910
67. Ritz E, Rambausek M, Mall G, Ruffmann K, Mandelbaum A. Cardiac changes in uraemia and their possible relationship to cardiovascular instability on dialysis. *Nephrol Dial Transplant*. 1990;5 Suppl 1:93-7. doi:10.1093/ndt/5.suppl_1.93
68. Marshall MR, Dunlop JL. Are dialysate sodium levels too high? *Semin Dial*. May 2012;25(3):277-83. doi:10.1111/j.1525-139X.2012.01072.x
69. Avkiran NK. An in-depth discussion and illustration of partial least squares structural equation modeling in health care. *Health Care Manag Sci*. Sep 2018;21(3):401-408. doi:10.1007/s10729-017-9393-7

70. Wieland A, Durach C, Kembro J, Treiblmaier H. Statistical and judgmental criteria for scale purification. *Supply Chain Management*. 08/01 2017;22(4):321-328. doi:10.1108/SCM-07-2016-0230
71. Ali F, Rasoolimanesh SM, Sarstedt M, Ringle CM, Ryu K. An assessment of the use of partial least squares structural equation modeling (PLS-SEM) in hospitality research. *International Journal of Contemporary Hospitality Management*. 2018;30(1):514-538. doi:10.1108/IJCHM-10-2016-0568
72. Dijkstra TK, Henseler J. Consistent partial least squares path modeling. *MIS Quarterly*. 2015;39(2):297-316.
73. Henseler J, Ringle CM, Sarstedt M. A new criterion for assessing discriminant validity in variance-based structural equation modeling. *Journal of the Academy of Marketing Science*. 2015/01/01 2015;43(1):115-135. doi:10.1007/s11747-014-0403-8
74. Hair JF, Hult GTM, Ringle CM, Sarstedt M. *A Primer on Partial Least Squares Structural Equation Modeling (PLS-SEM)*. 2nd ed. Sage Publications Inc.; 2017.
75. Sarstedt M, Hair Jr JF, Ringle CM. "PLS-SEM: indeed a silver bullet" – retrospective observations and recent advances. *Journal of Marketing Theory and Practice*. 2023/07/03 2023;31(3):261-275. doi:10.1080/10696679.2022.2056488
76. Hair Jr JF, Ringle CM, Sarstedt M. Partial least squares structural equation modeling: Rigorous applications, better results and higher acceptance. *Long Range Planning: International Journal of Strategic Management*. 2013;46:1-12. doi:10.1016/j.lrp.2013.01.001
77. Falk RF, Miller NB. *A primer for soft modeling*. 1st ed. Univeristy iof Akron Press; 1992.
78. Chin WW. The partial least squares approach to structural equation modeling. In: Marcoulides GA, ed. *Modern Methods for Business Research*. Lawrence Erlbaum Associates; 1998:295-336.
79. Cohen J. *Statistical power analysis for the behavioral sciences*. 2nd ed. Lawrence Erlbaum Associates; 1988.
80. Ringle CM, Sarstedt M, Schlittgen R. Finite Mixture and Genetic Algorithm Segmentation in Partial Least Squares Path Modeling: Identification of Multiple Segments in Complex Path Models. Springer Berlin Heidelberg; 2010:167-176.
